# Aromatic chloroosmacyclopentatrienes

**DOI:** 10.1093/nsr/nwac237

**Published:** 2022-10-28

**Authors:** Zhenwei Chu, Guomei He, Chuan Shi, Yuhui Hua, Yaxi Huang, Jiangxi Chen, Hujun Xie, Guochen Jia

**Affiliations:** Department of Materials Science and Engineering, College of Materials, Xiamen University, Xiamen 361005, China; Department of Materials Science and Engineering, College of Materials, Xiamen University, Xiamen 361005, China; Department of Chemistry, The Hong Kong University of Science and Technology, Hong Kong, China; Department of Chemistry, College of Chemistry and Chemical Engineering, Xiamen University, Xiamen 361005, China; Department of Materials Science and Engineering, College of Materials, Xiamen University, Xiamen 361005, China; Department of Materials Science and Engineering, College of Materials, Xiamen University, Xiamen 361005, China; Department of Applied Chemistry, Zhejiang Gongshang University, Hangzhou 310018, China; Department of Chemistry, The Hong Kong University of Science and Technology, Hong Kong, China

**Keywords:** metallacycles, chlorometallacyclopentatriene, osmium, *o*-ethynylphenyl alkynes, halocarbon complex

## Abstract

Aromatic metallacycles are of considerable current interest. Reported aromatic metallacycles are mainly those with carbon, nitrogen, oxygen and sulfur. In this work, we report the synthesis and characterization of aromatic chloroosmacyclopentatrienes, which represent the first structurally confirmed metallaaromatic with a chlorine atom in its framework. Single-crystal X-ray diffraction studies show that these planar chloroosmacyclopentatrienes possess a very short Os–ClC distance suggesting M=ClC bond character.

## Teaser

Metal-chlorine bonds of halocarbon complexes are usually longer than metal-halide bonds. By contrast, this work demonstrates that complexes LnOsCl(ClR) have the Os−ClR bond shorter than the Os−Cl bond.

## INTRODUCTION

**Figure 1.**
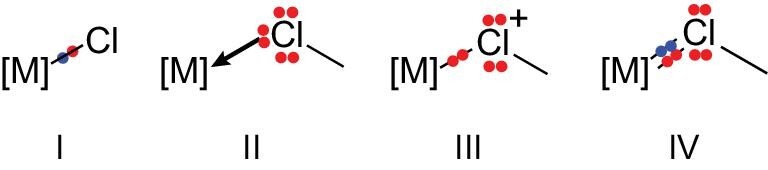
Types of bonding between transition metals and (**I**) chloride, (**II**–**IV**) chlorocarbons.

The chemical bond between an s-block metal and chlorine (such as LiCl or NaCl) is usually described as an ionic bond in textbooks [1]. Compared to s-block metals, d-block transition metals can have more types of bonding with chlorine. In addition to well-known metal halide complexes, which contain ionic or covalent σ M–Cl bonds (type **I** in Fig. 1), d-block transition metals can also form complexes with chlorocarbons (ClR). In coordination complexes with chlorocarbons, a dative M(η^1^-ClC) bond (type **II** in Fig. 1), in which the chlorocarbon serves as a 2e donating ligand, is commonly assumed [2]. A chlorine atom in chlorocarbons could also interact with d-block transition metals via other types of bonds, for example, the covalent type **III** in chloronium form [3] and the type **IV** containing a metal-chlorine double bond. The organic counterparts of the covalent type **III** bonding were reported a long time ago [4]. For example, electrophilic addition reactions of alkenes with Cl_2_ are known to occur via cyclochloronium intermediates. The covalent type **IV** bonding is closely related to the bonding in inorganic compounds such as FCl=O and Cl_3_CCl=O [5]. However, examples of organometallic versions of M(η^1^-Cl^+^C) (type **III**) and M=ClC (type **IV**) bonds are rarely reported.

Recently, the chemistry of compounds with an unusual carbon-halogen multiple bond is attracting attention both experimentally and theoretically. Novel compounds reported recently include compounds with a terminal C=Cl or C≡Cl bond [6], iodabenzene [7] and an η^3^-phenyldichloromethyl (η^3^-PhCCl_2_) complex [8]. Herein, we report the isolation and characterization of planar chlorometallacyclopentatrienes, in which the bond distance between the osmium atom and the ClC unit is very short, and the Os–ClC bond can be viewed as the M=ClC bond of type **IV**, possibly stabilized by the aromaticity of the metallacycle [9–24].

## RESULTS AND DISCUSSION

**Scheme 1.**
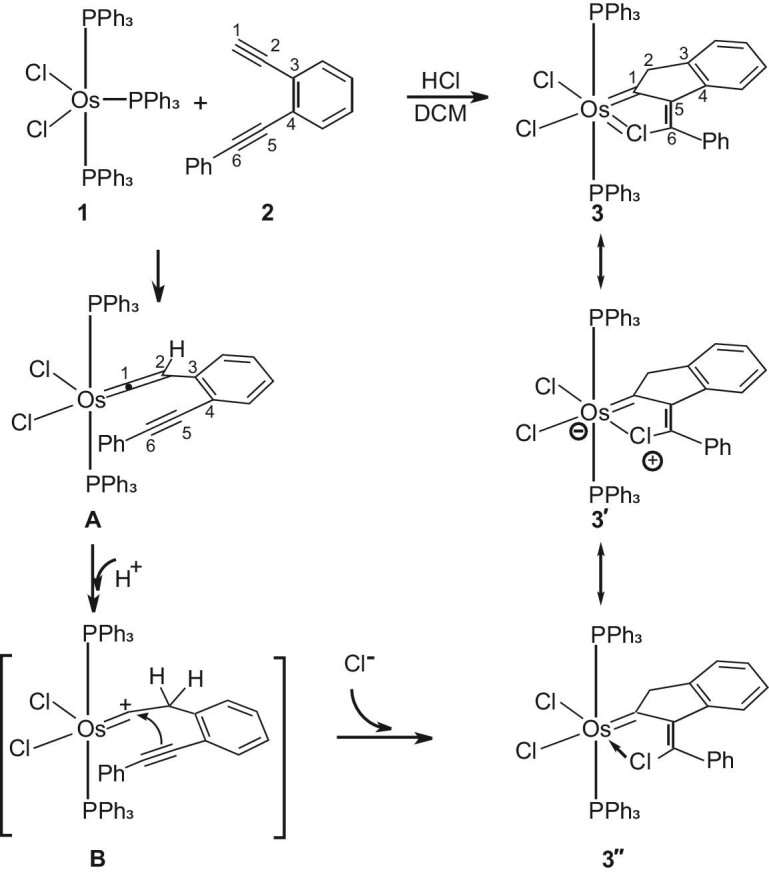
Osmium-mediated cyclization of an enediyne in the presence of HCl.

The first example of a chlorometallacyclopentatriene was isolated during our investigation of the reactivity of OsCl_2_(PPh_3_)_3_ (**1**) with the *o*-ethynylphenyl alkynes [25–28]. Thus, treatment of OsCl_2_(PPh_3_)_3_ (**1**) with 1-ethynyl-2-(phenylethynyl)benzene (**2**) and excess HCl in dichloromethane at room temperature for 3 h produced a green solution, from which the complex **3** was isolated as a green solid in 62.0% yield (Scheme 1).

**Figure 2.**
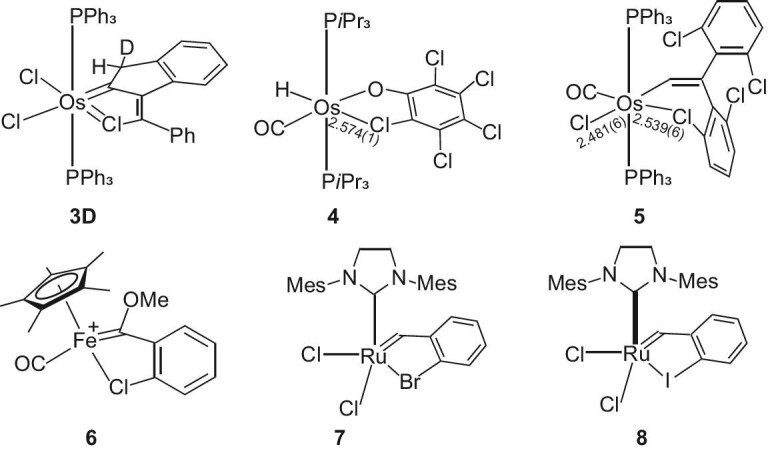
The structures of **3D** and **4**–**8**.

We propose that complex **3** might be formed via intermediates **A** and **B** as shown in Scheme 1. The reaction of OsCl_2_(PPh_3_)_3_ (**1**) with **2** could initially generate the vinylidene intermediate **A**. Reactions of OsCl_2_(PPh_3_)_3_ (**1**) with terminal alkynes (HC≡CR) to form osmium vinylidene complexes OsCl_2_(=C=CHR)(PPh_3_)_2_ are well-documented reactions [29–31]. It has also been reported that osmium vinylidene complexes can be protonated at their β-carbon to give osmium carbyne complexes [29–31]. Thus, the intermediate **A** could be protonated at the C2 position to give the intermediate **B**, which may undergo an electrophilic cyclization followed by the addition of Cl^−^ to give complex **3**. Consistent with the proposed mechanism, the reaction in the presence of DCl produced the partially deuterated complex **3D** (Fig. 2) with a CHD methylene group (see Figs S11–S13 for characterization data).

**Figure 3.**
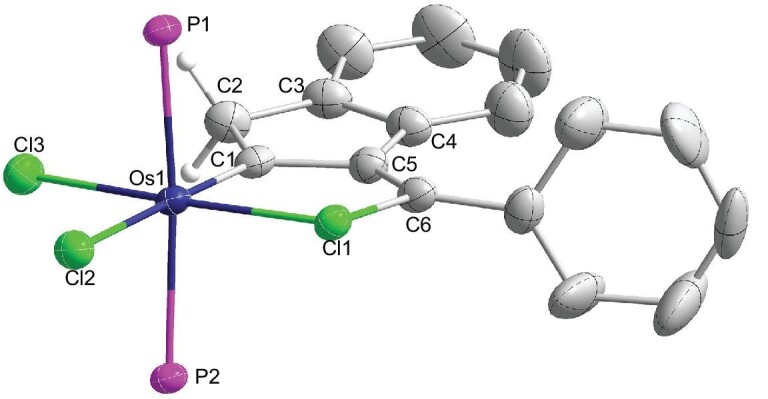
Single-crystal X-ray structure of complex **3** with thermal ellipsoids at the 50% probability level (phenyl groups in PPh_3_ are omitted for clarity). Selected bond distances (Å) and angles (°) in **3**: Os1–Cl3 2.3993(11), Os1–Cl2 2.5135(10), Os1–Cl1 2.3594(10), Os1–C1 1.903(4), C1-C2 1.515(6), C2-C3 1.495(6), C3-C4 1.397(7), C4-C5 1.480(6), C5-C6 1.336(6), C6-Cl1 1.781(4), C1-C5 1.498(6); Os1-C1-C5 121.7(3), C1-C5-C6 121.1(4), C5-C6-Cl1 113.2(3), C6-Cl1-Os1 101.28(15), Cl1-Os1-C1 82.61(13), C1-C2-C3 105.6(4), C2-C3-C4 111.3(4), C3-C4-C5 108.4(4), C4-C5-C1 108.5(4), C5-C1-C2 106.1(3).

The structure of complex **3** has been determined by single-crystal X-ray diffraction studies, which confirmed that complex **3** is a tricyclic metallacycle (Fig. 3). The five-membered osmacycle is almost planar, as reflected by the mean deviation (0.021 Å) from the least-squares plane of Os1, C1, C5, C6 and Cl1. The Os–C1 bond length of 1.903(4) Å is in the range of reported Os=CHR lengths (1.810–2.142 Å, based on a search of the Cambridge Structural Database, CSD version 2020 (Nov 2019)) [32–36], which suggests its double-bond character. The most fascinating structural feature in metallacycle **3** is that the distance of Os–Cl1 (2.359 Å) is even shorter (by 0.040 Å) than that of the terminal Os–Cl3 bond (2.399 Å), indicating the unusual bonding (beyond a σ-bonding) between Os and Cl. It is noted that there are only two X-ray single-crystal structures (complexes **4** and **5** shown in Fig. 2) containing short Os···ClC contacts in CSD version 2020 (Nov 2019). In these two examples, the bond lengths of Os–ClAr are 2.574(1) Å for **4** [37] and 2.539(6) Å for **5** [38], which are typical dative bond lengths. Thus, complex **3** represents an interesting example that contains an unusually short Os–ClC bond. Notably, all the CSD crystal structures that contain both M–ClC and terminal M–Cl (M: transition metal) bonds have a distance of an M–ClC longer than that of a terminal M–Cl.

Furthermore, the distance of Cl1–C6 (1.781(4) Å) is longer than those of Cl–C(*sp^2^*) bonds in dative ClC unit (1.739 Å for **4** and 1.754 Å for **5**) and longer than those of organic Cl–C(*sp*^2^) bonds (e.g. 1.712–1.747 Å for **4** and **5**). Moreover, the bond lengths of C5–C6 (1.336(6) Å) and C1–C5 (1.498(6) Å) in **3** are in the range of C–C single (1.54 Å) and double (1.33 Å) bond distances. These results suggest that a weak delocalization is present in the chlorometallacycle ring of complex **3**. Previously reported structurally characterized complexes that are closely related to complex **3** include [Cp*Fe(CO){*k*^2^-C(OMe)C_6_H_4_-*o*-Cl}]OTf (**6**) and RuCl_2_(*k*^2^-CH-Ar-*o*-X)(IMes) (**7**, X = Br; **8**, X = I) [39–42]. The five-membered chlorometallacycle in **6** does not however adopt a planar structure [39]. The complexes **7** (X = Br) and **8** (X = I) have longer Ru–XAr (X = Br, I) bond distances (longer than 2.5007 Å) [40–42], which are typical dative bonds. Thus, the structural features of complex **3** are completely different from those of complexes **6**–**8**.

The nuclear magnetic resonance (NMR) spectroscopy and high-resolution mass spectroscopy (HRMS) data of **3** are consistent with the solid structure. For instance, complex **3** shows the signal of proton (C2*H*) at 0.57 ppm in the ^1^H NMR spectrum and the signal of *C*2 at 62.0 ppm in the ^13^C{^1^H} NMR spectrum, confirming that *C*2 has an *sp*^3^ hybridized character. The signal of metal-bonded *C*1 is located at 271.6 ppm in the ^13^C{^1^H} NMR spectrum, supporting the metal-carbene character of *C*1. Only one sharp signal appeared at -19.4 ppm in the ^31^P{^1^H} NMR spectrum, indicating the equivalent chemical environment of the two *trans* PPh_3_ ligands, which confirms the planar structure of fused rings. The molecular formula of complex **3** was also confirmed by HRMS data (m/z = 989.1647 for [**3**-**Cl**]^+^).

As shown in scheme 1, there are three possible resonance forms that could contribute to the overall structure of complex **3**: the neutral metallacycle **3**, the zwitterionic form **3’** and the chloride dative form **3”**. The resonance form **3”** cannot well account for the fact that the distance of Os–Cl1 (2.359 Å) in the metallacycle is much shorter than those of dative Os–ClAr bonds (2.574 Å for **4** and 2.538 Å for **5**) and even shorter than that of Os–Cl3 (2.399 Å, a typical metal chloride σ-bond). The short Os–ClC distance in **3** suggests the M–ClC has a double-bond character, which can be attributed to the contribution from the resonance form **3**. Complex **3** can be regarded as a chlorometallacyclopentatriene, which is a metal analog of chlorophenium ([C_4_H_4_Cl]^+^). Chlorophenium ([C_4_H_4_Cl]^+^) is closely related electronically to five-membered heteroaromatics such as thiophene or furan.

**Figure 4.**
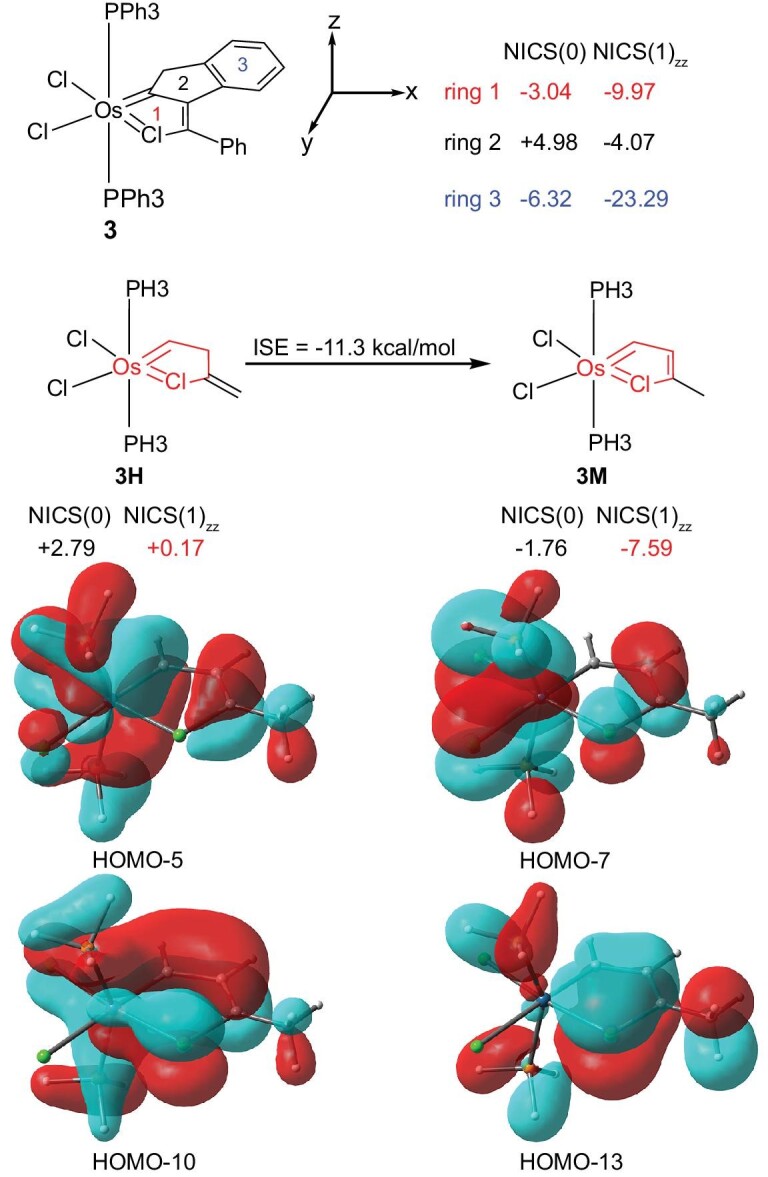
NICS(0) and NICS(1)_zz_ values for complexes **3, 3H** and **3M**; the isomerization stabilization energy of **3M** from **3H**; key occupied π-MOs of **3M** (the isosurface value is 0.02).

To check whether the chloroosmacyclopentatriene is aromatic or not, we calculated the nucleus-independent chemical shift (NICS) value [43], an index used to evaluate aromaticity or anti-aromaticity. The structure of **3** was optimized at PBE/def2-TZVP level [44,45] and the value of NICS(1)_zz_ of the chlorometallacycle ring in complex **3** was calculated (at PBE/x2c-TZVPall level [44,46]) to be -9.97 (Fig. 4), suggesting that chlorometallacycle **3** has an aromatic character.

To further understand the aromaticity of **3**, we have performed a theoretical study of the model complex OsCl_2_(PH_3_)_2_(*k*^2^-ClC_3_H_2_Me) (**3M**, see Fig. 4 for its structure) at PBE/def2-TZVP level [44,45]. The optimized structure of **3M** well reproduced the key structural feature of **3**. The aromatic character of the chloroosmacyclopentatriene **3M** is indicated by the calculated isomerization stabilization energy of -11.3 kcal/mol (the energy involved in the isomerization of **3H** to form **3M**, see Fig. 4). Moreover, the negative NICS(1)_zz_ value (−7.59, calculated at PBE/x2c-TZVPall level [44,46] based on the optimized structure) of **3M** also supports the aromaticity of the chloroosmacyclopentatriene.

As shown in Fig. 4, there are a total of four key occupied π-type molecular orbitals (HOMO-5, HOMO-7, HOMO-10, HOMO-13, see Fig. 4) for the metallacycle of **3M**, indicating that the chlorometallacycle has eight π electrons. Thus, this planar chlorometallacyclopentatriene **3** is a unique Möbius [17,19,22,47–50] aromatic complex. It is worth noting that the π-bonding molecular orbitals of chlorometallacycle are generated by interactions of two d orbitals of an OsX_2_L_2_ fragment with the π-type molecular orbitals of a ClC_3_H_2_Me fragment. In other words, osmium can form π-bonds with the carbon-bound chlorine atom by back-donation from the osmium d orbitals to the π-type molecular orbitals of the ClC_3_H_2_Me fragment. As a result, the unusually short M–ClC bond in **3** can be partially related to a *d*_π_-*p*_π_ π-interaction between the osmium atom and the ClC unit, as indicated by the resonance form **3**. Thus, chloroosmacyclopentatriene **3** represents the first structurally confirmed aromatic chlorometallacycle with an M=Cl–C character.

**Scheme 2.**
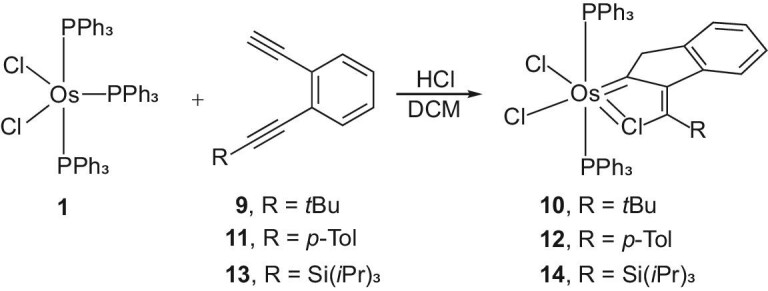
The preparation of chloroosmacyclopentatrienes.

The unusual structural feature of complex **3** prompted us to synthesize its analogues. By reacting *o*-ethynylphenyl alkynes **9, 11** and **13** with OsCl_2_(PPh_3_)_3_ (**1**), the chloroosmacyclopentatriene derivatives **10, 12** and **14** with a *t*Bu, *p*-Tol or Si(*i*Pr)_3_ (TIPS) substituent, respectively, were obtained (Scheme 2).

**Figure 5.**
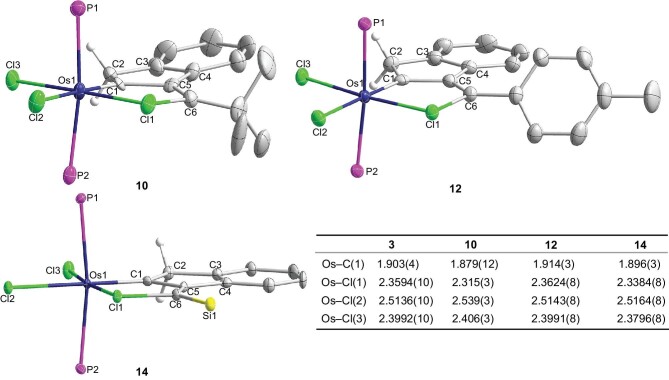
Single-crystal X-ray structures of **10, 12** and **14** with thermal ellipsoids at 50% probability level (the phenyl groups in PPh_3_ and the isopropyl groups in the Si(*i*Pr)_3_ are omitted for clarity), and selected bond distances (Å) for complexes **3, 10, 12** and **14**.

The complexes **10, 12** and **14** were fully characterized by NMR spectroscopy and X-ray diffraction studies (Fig. 5). They all have structural features similar to those in **3**, in which the M–ClR bond is systematically shorter than the corresponding M–Cl(terminal) bond. Subtle differences in the structures of complexes **3, 10, 12** and **14** are noted (Fig. 5). The Os–ClR bond in *p*-Tol-substituted osmium complex **12** (2.3624(8) Å) is similar to that of Ph-substituted osmium complex **3** (2.3594(10) Å), and an appreciably shorter Os–ClR bond is observed for *t*Bu-substituted complex **10** (2.315(3) Å) and TIPS-substituted osmium complex **14** (2.3384(8) Å). The difference in the Os–Cl1(chlorocarbon) and Os–Cl3(terminal) bond distances in *t*Bu-substituted complex **10** is as large as 0.091 Å.

## CONCLUSION

In summary, OsCl_2_(PPh_3_)_3_ was found to react with *o*-ethynylphenyl alkynes in the presence of HCl to give chlorometallacyclopentatriene complexes. These complexes are unusual in that the M–ClC(halocarbon) bond is appreciably shorter than the M–Cl(chloride) bond, and they can be regarded as compounds possessing an M=ClC bond. The novel metallacycles exhibit aromaticity, excellent planarity and thermal stability. This study not only enriches the family of metallacyclic chemistry, but also provides the synthesis of novel metallacycles containing an M=ClC bond.

## Supplementary Material

nwac237_Supplemental_File
