# Supplementary material for: Aromatic chloroosmacyclopentatrienes
Source: Natl Sci Rev. 2022 Oct 28;10(3):nwac237. doi: 10.1093/nsr/nwac237 (PMC10103825; doi:10.1093/nsr/nwac237)
Supplement: nwac237_Supplemental_File [file nwac237_supplemental_file.docx]

Aromatic chlorometallacyclopentatrienes

Zhenwei Chu^1^, Guomei He^1^, Chuan Shi^3^, Yuhui Hua^4^, Yaxi Huang^1^, Jiangxi Chen^1*^, Hujun Xie^2*^, Guochen Jia^3*^

^1^Department of Materials Science and Engineering, College of Materials, Xiamen University, Xiamen 361005, China.

^2^Department of Applied Chemistry, Zhejiang Gongshang University, Hangzhou 310018, China.

^3^Department of Chemistry, The Hong Kong University of Science and Technology, Hong Kong, China.

^4^Department of Chemistry, College of Chemistry and Chemical Engineering, Xiamen University, Xiamen 361005, China.

*Correspondence and requests for materials should be addressed to J.C. (email: chenjx@xmu.edu.cn), H.X. (e-mail: hjxie@zjgsu.edu.cn), G.J. (e-mail: chjiag@ust.hk).

Table of Contents

1. Supplemental Experimental Procedures S1

2. X-ray Crystallographic Analysis S2

3. NMR and HRMS Spectra S5

4. Computational Details S17

5. The Calculated Cartesian Coordinates with Electronic Energies S17

6. Supplemental References S18

1. Supplemental Experimental Procedures

**General Information:**

All manipulations were carried out under a nitrogen atmosphere using standard Schlenk techniques unless otherwise stated. Solvents were distilled under nitrogen from sodium benzophenone (diethyl ether, benzene, hexane), or calcium hydride (CH_2_Cl_2_). Reagents were used as received from commercial sources without further purification. OsCl_2_(PPh)_3_ (1) [1], 1-ethynyl-2-(phenylethynyl)benzene (2) [2], 1-(3,3-dimethylbut-1-yn-1-yl)-2-ethynylbenzene (9) [2], 1-ethynyl-2-(*p*-tolylethynyl)benzene (11) [3], and ((2-ethynylphenyl)ethynyl)triisopropylsilane (13) [4] were prepared according to the previously published procedure. ^1^H, ^13^C{^1^H}, and ^31^P{^1^H} spectra were collected on a Bruker Advance II-300 spectrometer (300 MHz), or a Bruker AVIII-400 spectrometer (400 MHz), or a Bruker AVIII-500 spectrometer (500 MHz), or a Bruker Ascend III 600 spectrometer at room temperature. ^1^H and ^13^C NMR shifts are relative to TMS, and ^31^P chemical shifts relative to 85% H_3_PO_4_. Two-dimensional and one-dimensional NMR spectra are abbreviated as HMBC (heteronuclear multiple bond coherence) and DEPT (distortionless enhancement by polarization transfer). The absolute values of the coupling constants are given in hertz (Hz). Multiplicities are abbreviated as s (singlet), d (doublet), t (triplet), q (quartet), m (multiplet) and br (broad). High resolution mass spectra (HRMS) experiments were recorded on a Bruker FT-MS. Elemental analyses were performed on a Vario EL III elemental analyzer.

**Complex 3.** To a solution of OsCl_2_(PPh)_3_ (**1**) (1.029 g, 0.98 mmol) in dichloromethane (10 mL) was added 1-ethynyl-2-(phenylethynyl)benzene (**2**) (0.298 g, 1.48 mmol) and then hydrogen chloride (2.0 M in diethyl ether, 3.0 mL, 6.0 mmol). The mixture was stirred at room temperature for 3 h to give a green solution. Then, the solvent of the reaction mixture was removed under vacuum. The residue was extracted with benzene (10 mL × 2) to give a green solution and a precipitate, which was separated by filtration. All the solvent of the extract was removed under vacuum, and the residue was washed with diethyl ether (10 mL × 2) to give a green precipitate, which was collected by filtration and dried under vacuum. Yield, 623 mg, 62.0 %. ^31^P{^1^H} NMR (121.5 MHz, CD_2_Cl_2_): *δ* -19.4 (s). ^1^H NMR (300.1 MHz, CD_2_Cl_2_): *δ* 0.57 (s, 2 H, H2), 6.86 (t, *J*_HH_ = 7.8 Hz, 3 H, *Ph*), 6.91 (d, *J*_HH_ = 7.8 Hz, 1 H, *Ph*), 7.02 (t, *J*_HH_ = 7.4 Hz, 1 H, *Ph*), 7.08 (d, *J*_HH_ = 6.9 Hz, 1 H, *Ph*), 7.12-7.31 (m, 18 H, P*Ph*_3_), 7.39 (t, *J*_HH_ = 7.7 Hz, 2 H, *Ph*), 7.51 (t, *J*_HH_ = 7.5 Hz, 1 H, *Ph*), 7.54-7.64 (m, 12 H, P*Ph*_3_). ^13^C{^1^H} NMR (150.9 MHz, CD_2_Cl_2_): *δ* 62.0 (s, C2), 124.0 (s, *Ph*), 125.9 (s, C7), 126.1 (s, C9), 128.2 (t, *J*_PC_ = 4.6 Hz, P*Ph*_3_), 128.9 (s, C10), 129.7 (s, C8), 130.5 (s, P*Ph*_3_), 130.7 (s, *Ph*), 131.5 (s, *Ph*), 132.6 (t, *J*_PC_ = 24.7 Hz, P*Ph*_3_), 134.2 (s, C4), 134.9 (s, *Ph*), 135.3 (t, *J*_PC_ = 4.9 Hz, P*Ph*_3_), 150.8 (s, C3), 155.0 (s, C6), 161.5 (s, C5), 271.6 (t, *J*_PC_ = 9.1 Hz, C1). HRMS (ESI) m/z calcd for [C_52_H_41_Cl_2_OsP_2_]^＋^: 989.1670, found: 989.1647. Anal. Calcd (%) for C_52_H_41_Cl_3_OsP_2_: C, 60.97; H, 4.03. Found: C, 60.83; H, 3.85.

**Complex 3D.** To a solution of OsCl_2_(PPh)_3_ **(1)** (1.009 g, 0.96 mmol) in dichloromethane (10 mL) was added 1-ethynyl-2-(phenylethynyl)benzene **(2)** (0.303 g, 1.50 mmol) and then deuterium chloride (1.0 M in diethyl ether, 5.7 mL, 5.7 mmol). The mixture was stirred at room temperature for 3 h to give a green solution. Then, the solvent of the reaction mixture was removed under vacuum. The residue was extracted with benzene (10 mL×2) to give a green solution and a precipitate, which was separated by filtration. All the solvent of the extract was removed under vacuum, and the residue was washed with diethyl ether (10 mL×2) to give a green precipitate, which was collected by filtration and dried under vacuum. Yield, 628 mg, 63.8 %.^2^H NMR (400.0 MHz, CH_2_Cl_2_): *δ*  0.56 (s, CH*D*). HRMS (ESI) m/z calcd for [C_52_H_40_DCl_2_OsP_2_]^＋^: 990.1733 found: 990.1728.

**Complex 10.** To a solution of OsCl_2_(PPh)_3_ (**1**) (0.102 g, 0.097 mmol) in dichloromethane (2.0 mL) was added 1-(3,3-dimethylbut-1-yn-1-yl)-2-ethynylbenzene (**9**) (0.027 g, 0.148 mmol) and then hydrogen chloride (2.0 M in diethyl ether, 0.30 mL, 0.60 mmol). The mixture was stirred at room temperature for 3 h to give a green solution. Then, the solvent of the reaction mixture was removed under vacuum. The residue was added diethyl ether (0.3 mL × 3) to give a green precipitate, which was collected by filtration and dried under vacuum. Yield, 82 mg, 84.2 %. ^31^P{^1^H} NMR (121.5 MHz, CD_2_Cl_2_): *δ* -20.6 (s). ^1^H NMR (300.1 MHz, CD_2_Cl_2_): *δ* 0.54 (s, 2 H, H2), 1.14 (s, 9 H, H12), 6.84 (d, *J*_HH_ = 7.5 Hz, 1 H, *Ph*), 7.12-7.28 (m, 20 H, *Ph*), 7.46-7.53 (m, 12 H, P*Ph_3_*), 7.68 (d, *J*_HH_ = 8.1 Hz, 1 H, *Ph*). ^13^C{^1^H} NMR (150.9 MHz, CD_2_Cl_2_): *δ* 28.5 (s, C12), 38.8 (s, C11), 62.1 (s, C2), 125.9 (s, C7), 126.1 (s, C9), 128.2 (t, *J*_PC_ = 4.8 Hz, P*Ph*_3_), 128.7 (s, C10), 129.6 (s, C8), 130.4 (s, P*Ph*_3_), 132.2 (t, *J*_PC_ = 24.6 Hz, P*Ph*_3_), 134.2 (s, C4), 135.3 (t, *J*_PC_ = 4.4 Hz, P*Ph*_3_), 151.4 (s, C3), 158.7 (s, C5), 172.8 (s, C6), 275.7 (t, *J*_PC_ = 8.6 Hz, C1). HRMS (ESI) m/z calcd for [C_50_H_45_Cl_2_OsP_2_]^＋^: 969.1983, found: 969.1986. Anal. Calcd (%) for C_50_H_45_Cl_3_OsP_2_: C, 59.79; H, 4.52. Found: C, 59.89; H, 4.75.

**Complex 12.** To a solution of OsCl_2_(PPh)_3_ (**1**) (0.500 g, 0.48 mmol) in dichloromethane (10 mL) was added 1-ethynyl-2-(*p*-tolylethynyl)benzene (**11**) (0.154 g, 0.71 mmol) and then hydrogen chloride (2.0 M in diethyl ether, 1.5 mL, 3.0 mmol). The mixture was stirred at room temperature for 3 h to give a green solution. Then, the solvent of the reaction mixture was removed under vacuum. The residue was extracted with benzene (5 mL × 2) to give a green solution and a precipitate, which was separated by filtration. All the solvent of the extract was removed under vacuum, and the residue was washed with diethyl ether (3 mL × 3) to give a green precipitate, which was collected by filtration and dried under vacuum. Yield, 322 mg, 64.6 %. ^31^P{^1^H} NMR (121.5 MHz, CD_2_Cl_2_): *δ* -22.8 (s). ^1^H NMR (300.1 MHz, CD_2_Cl_2_): *δ* 0.57 (s, 2 H, H2), 2.43 (s, 3 H, H15), 6.74 (d, *J*_HH_ = 7.5 Hz, 2 H, *Ph*), 6.87 (d, *J*_HH_ = 6.9 Hz, 1 H, *Ph*), 6.95 (d, *J*_HH_ = 6.9 Hz, 1 H, *Ph*), 6.99-7.08 (m, 2 H, *Ph*), 7.14-7.29 (m, 20 H, *Ph*), 7.53-7.65 (m, 12 H, *Ph*). ^13^C{^1^H} NMR (150.9 MHz, CD_2_Cl_2_): *δ* 22.1 (s, C15), 62.0 (s, C2), 124.0 (s, C12), 125.9 (s, C7), 126.1 (s, C9), 128.2 (t, *J*_PC_ = 4.3 Hz, P*Ph*_3_), 128.8 (s, C10), 130.3 (s, C13), 130.5 (s, P*Ph*_3_), 130.6 (s, C8), 131.9 (s, C11), 132.7 (t, *J*_PC_ = 24.5 Hz, P*Ph*_3_), 134.4 (s, C4), 135.3 (t, *J*_PC_ = 4.3 Hz, P*Ph*_3_), 142.3 (s, C14), 150.7 (s, C3), 155.5 (s, C6), 161.0 (s, C5), 271.7 (t, *J*_PC_ = 9.1 Hz, C1). HRMS (ESI) m/z calcd for [C_53_H_43_Cl_2_OsP_2_]^＋^: 1003.1826, found: 1003.1824. Anal. Calcd (%) for C_53_H_43_Cl_3_OsP_2_: C, 61.30; H, 4.17. Found: C, 61.40; H, 4.53.

**Complex 14.** To a solution of OsCl_2_(PPh)_3_ (**1**) (0.601 g, 0.57 mmol) in dichloromethane (10 mL) was added ((2-ethynylphenyl)ethynyl)triisopropylsilane (**13**) (0.248 g, 0.88 mmol) and then hydrogen chloride (2.0 M in diethyl ether, 1.7 mL, 3.4 mmol). The mixture was stirred at room temperature for 3 h to give a green solution. Then, the solvent of the reaction mixture was removed under vacuum. The residue was washed with diethyl ether (2 mL × 3) to give a green precipitate, which was collected by filtration and dried under vacuum. Yield, 427 mg, 67.5%. ^31^P{^1^H} NMR (161.9 MHz, CD_2_Cl_2_): *δ* -19.4 (s). ^1^H NMR (400.0 MHz, CD_2_Cl_2_): *δ* 0.35 (s, 2 H, H2), 0.86 (d, *J*_HH_ = 7.2 Hz, 18 H, H12), 1.45 (dt, *J*_HH_ = 15.2 Hz, *J*_HH_ = 14.8 Hz 3 H, H11), 6.77 (d, *J*_HH_ = 8.0 Hz, 1 H, *Ph*), 7.07-7.12 (m, 12 H, P*Ph_3_*), 7.21-7.22 (m, 6 H, P*Ph_3_*), 7.30-7.35 (m, 2 H, *Ph*), 7.55-7.57 (m, 12 H, P*Ph_3_*), 7.89-8.00 (m, 1 H, *Ph*). HRMS (ESI) m/z calcd for [C_55_H_57_Cl_2_OsP_2_]^＋^: 1069.2691 found: 1069.2783.

2. X-ray Crystallographic Analysis

All single crystals suitable for X-ray diffraction were grown from dichloromethane solution layered with hexane, Single-Crystal X-ray diffraction data of **3**, **10** and **12** were collected on an Oxford Gemini S Ultra CCD area detector using graphite-monochromated Mo Kα radiation (λ = 0.71073 Å). The diffraction intensity data of **14** was collected on a Rigaku Oxford Diffraction SuperNova, XtaLAB Synergy Dualflex HyPix area detector with monochromated Cu Kα radiation (λ =1.54184 Å). Using Olex2 [5], the structures of **3**, **10**, **12** and **14** were solved with the ShelXT [6] structure solution program using Intrinsic Phasing and refined with the ShelXL [7] refinement package using least-squares minimization. All non-hydrogen atoms were refined anisotropically unless otherwise stated. Hydrogen atoms were placed at idealized positions and assumed the riding model. Some of the solvent molecules and phenyl groups were disordered and refined with suitable restraints. The X-ray crystal structures have been deposited in the Cambridge Crystallographic Data Centre under the deposition numbers CCDC-1897528 (**complex 3**), CCDC-1897526 (**complex 10**), CCDC-1897527 (**complex 12**), CCDC-1897529 (**complex 14**). The data can be obtained free of charge from the CCDC (www.ccdc.cam.ac.uk/data_request/cif).

**Crystal Data** **for 3:** C_54.5_H_46_Cl_8_OsP_2_ C_52_H_41_Cl_3_OsP_2_•2.5CH_2_Cl_2_ (*Mr* =1236.65 g/mol): triclinic, crystal dimensions of 0.3 × 0.2 × 0.2 mm, space group P_-1_ (no. 2), *a* = 12.1930(5) Å, *b* = 13.4317(5) Å, *c* = 17.6473(7) Å, *α* = 108.854(4) °, *β* = 95.073(3)°, *γ* = 107.757(4)°, *V* = 2548.10(19) Å^3^, *Z* = 2, *T* = 293(2) K, *μ*(MoKα) = 3.022 mm^-1^, *Dcalc* = 1.612 g/cm^3^, 20906 reflections measured (5.966° ≤ 2Θ ≤ 54.998°), 11683 unique (*R*_int_ = 0.0377, *R*_sigma_ = 0.0561) which were used in all calculations. The final *R*_1_ was 0.0388 (I > 2σ(I)) and *wR*_2_ was 0.0933 (all data), GOF = 1.054. Residual electron density (e. Å^-3^) max/min: 1.50/-1.32.

**Crystal Data for 10:** C_52.5_H_49_Cl_8_OsP_2_ C_50_H_45_Cl_3_OsP_2_•2.5CH_2_Cl_2_ (*Mr* =1215.65 g/mol): monoclinic, crystal dimensions of 0.3 × 0.2 × 0.2 mm, space group P2_1_/n (no. 14), *a* = 13.6433(8) Å, *b*= 19.3291(8) Å, *c* = 19.9314(11) Å, *β* = 88.201(5)°, *V* = 5253.6(5) Å^3^, *Z* = 4, *T* = 293(2) K, *μ*(MoKα) = 2.930 mm^-1^, *Dcalc* = 1.537 g/cm^3^, 13737 reflections measured (5.976° ≤ 2Θ ≤ 49.998°), 13737 unique (*R*_int_ = merged , *R*_sigma_ = 0.1083) which were used in all calculations. The final *R*_1_ was 0.0774 (I > 2σ(I)) and *wR*_2_ was 0.2064 (all data). GOF = 1.017. Residual electron density (e. Å^-3^) max/min: 3.27/-2.50.

**Crystal Data for 12:** C_56_H_48_Cl_9_OsP_2_ C_53_H_43_Cl_3_OsP_2_•3CH_2_Cl_2_ (*Mr* =1292.13 g/mol): triclinic, crystal dimensions of 0.5 × 0.4 × 0.4 mm, space group P_-1_ (no. 2), *a* = 13.0829(4) Å, *b* = 14.2440(5) Å, *c* = 14.9793(5) Å, *α* = 87.122(3)°, *β* = 76.273(3)°, *γ* = 84.955(3)°, *V* = 2699.96(16) Å^3^, *Z* = 2, *T* = 293(2) K, *μ*(MoKα) = 2.903 mm^-1^, *Dcalc* = 1.589 g/cm^3^, 23554 reflections measured (6.254° ≤ 2Θ ≤ 54.998°), 12381 unique (*R*_int_ = 0.0278, *R*_sigma_ = 0.0419) which were used in all calculations. The final *R*_1_ was 0.0324 (I > 2σ(I)) and *wR*_2_ was 0.0769 (all data). GOF = 1.050. Residual electron density (e. Å^-3^) max/min: 1.70/-1.73.

**Crystal Data for 14:** C_55_H_57_Cl_3_OsP_2_Si C_55_H_57_Cl_3_OsP_2_Si (*Mr* =1104.58 g/mol): monoclinic, crystal dimensions of 0.2 × 0.2 × 0.2 mm space group P2_1_/m (no. 11), *a* = 10.92610(10) Å, *b* = 22.32530(10) Å, *c* = 11.09380(10) Å, *β* = 119.0390(10)°, *V* = 2365.91(4) Å^3^, *Z* = 2,*T* = 100.00(10) K, *μ*(CuKα) = 7.814 mm^-1^, *Dcalc* = 1.551 g/cm^3^, 33583 reflections measured (7.92° ≤ 2Θ ≤ 129.988°), 4132 unique (*R*_int_ = 0.0337, *R*_sigma_ = 0.0179) which were used in all calculations. The final *R*_1_was 0.0212 (I > 2σ(I)) and *wR*_2_ was 0.0496 (all data). GOF = 1.128. Residual electron density (e. Å^-3^) max/min: 0.57/-1.16.

**Figure S1.** ORTEP drawing of **3** with thermal ellipsoids at 50% probability level. The phenyl groups on the PPh_3_ ligands are omitted for clarity. Selected bond lengths [Å] and angles [°]: Os1–Cl3 2.3993(11), Os1–Cl2 2.5135(10), Os1–Cl1 2.3594(10), Os1–C1 1.903(4), C1-C2 1.515(6), C2-C3 1.495(6), C3-C4 1.397(7), C4-C5 1.480(6), C5-C6 1.336(6), C6-Cl1 1.781(4), C1-C5 1.498(6); Os1-C1-C5 121.7(3), C1-C5-C6 121.1(4), C5-C6-Cl1 113.2(3), C6-Cl1-Os1 101.28(15), Cl1-Os1-C1 82.61(13), C1-C2-C3 105.6(4), C2-C3-C4 111.3(4), C3-C4-C5 108.4(4), C4-C5-C1 108.5(4), C5-C1-C2 106.1(3).

**
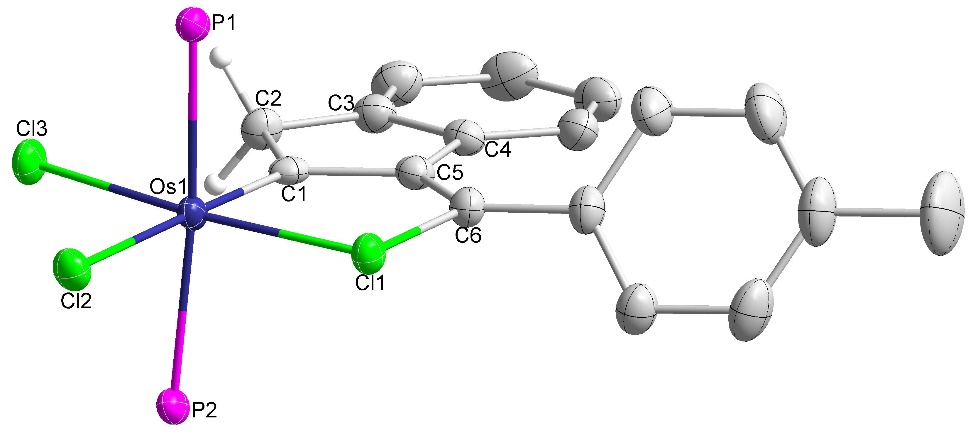
**
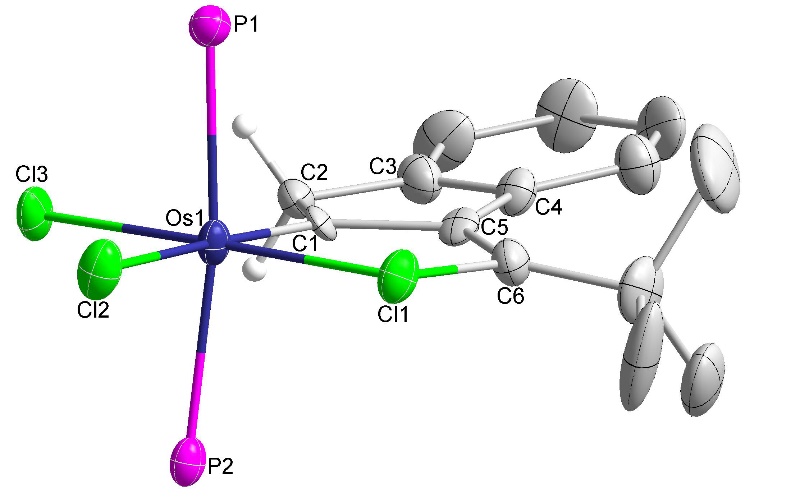
**Figure S2.** ORTEP drawing of **10** with thermal ellipsoids at 50% probability level. The phenyl groups on the PPh_3_ ligands are omitted for clarity. Selected bond lengths [Å] and angles [°]: Os1–Cl3 2.406(3), Os1–Cl2 2.539(3), Os1–Cl1 2.315(3), Os1–C1 1.879(12), C1-C2 1.512(16), C2-C3 1.493(17), C3-C4 1.404(18), C4-C5 1.470(17), C5-C6 1.370(17), C6-Cl1 1.771(13), C1-C5 1.493(16); Os1-C1-C5 123.5(8), C1-C5-C6 119.0(11), C5-C6-Cl1 111.6(9), C6-Cl1-Os1 103.3(4), Cl1-Os1-C1 82.1(3), C1-C2-C3 105.7(10), C2-C3-C4 111.9(12), C3-C4-C5 106.8(11), C4-C5-C1 110.2(10), C5-C1-C2 105.1(10).

**
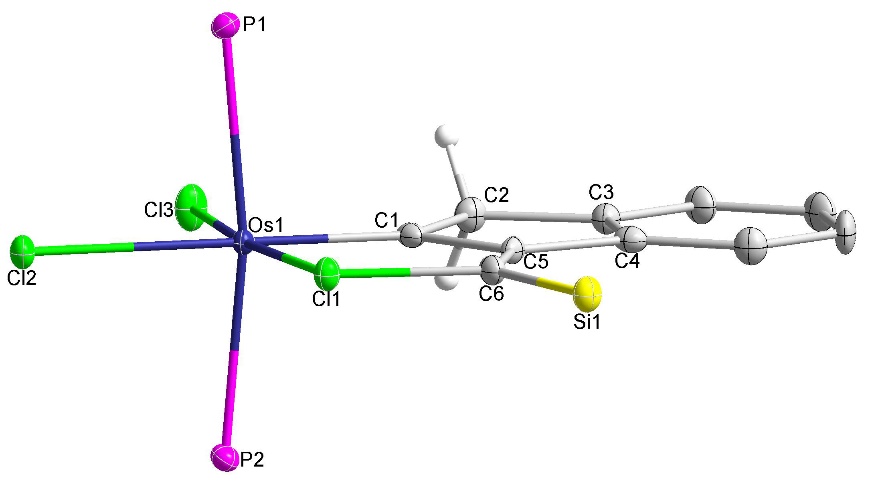
Figure S3.** ORTEP drawing of **12** with thermal ellipsoids at 50% probability level. The phenyl groups on the PPh_3_ ligands are omitted for clarity. Selected bond lengths [Å] and angles [°]: Os1–Cl3 2.3991(8), Os1–Cl2 2.5143(8), Os1–Cl1 2.3624(8), Os1–C1 1.914(3), C1-C2 1.515(4), C2-C3 1.502(5), C3-C4 1.399(5), C4-C5 1.476(5), C5-C6 1.352(5), C6-Cl1 1.790(3), C1-C5 1.480(5); Os1-C1-C5 122.7(2), C1-C5-C6 120.8(3), C5-C6-Cl1 112.7(2), C6-Cl1-Os1 101.56(11), Cl1-Os1-Cl1 82.05(10), C1-C2-C3 105.5(3), C2-C3-C4 110.7(3), C3-C4-C5 108.4(3), C4-C5-C1 109.0(3), C5-C1-C2 106.3(3).

**Figure S4.** ORTEP drawing of **14** with thermal ellipsoids at 50% probability level. The phenyl groups on the PPh_3_ ligands and the isopropyl groups on the Si(*i*Pr)_3_ are omitted for clarity. Selected bond lengths [Å] and angles [°]: Os1–Cl3 2.3796(8), Os1–Cl2 2.5164(8), Os1–Cl1 2.3384(8), Os1–C1 1.896(3), C1-C2 1.515(5), C2-C3 1.507(5), C3-C4 1.404(5), C4-C5 1.487(5), C5-C6 1.354(5), C6-Cl1 1.831(4), C1-C5 1.498(5); Os1-C1-C5 123.6(3), C1-C5-C6 122.5(3), C5-C6-Cl1 108.4(3), C6-Cl1-Os1 104.14(12), C1-Os1-Cl1 81.33(11), C1-C2-C3 105.4(3), C2-C3-C4 111.1(3), C3-C4-C5 108.5(3), C4-C5-C1 108.2(3), C5-C1-C2 106.8(3).


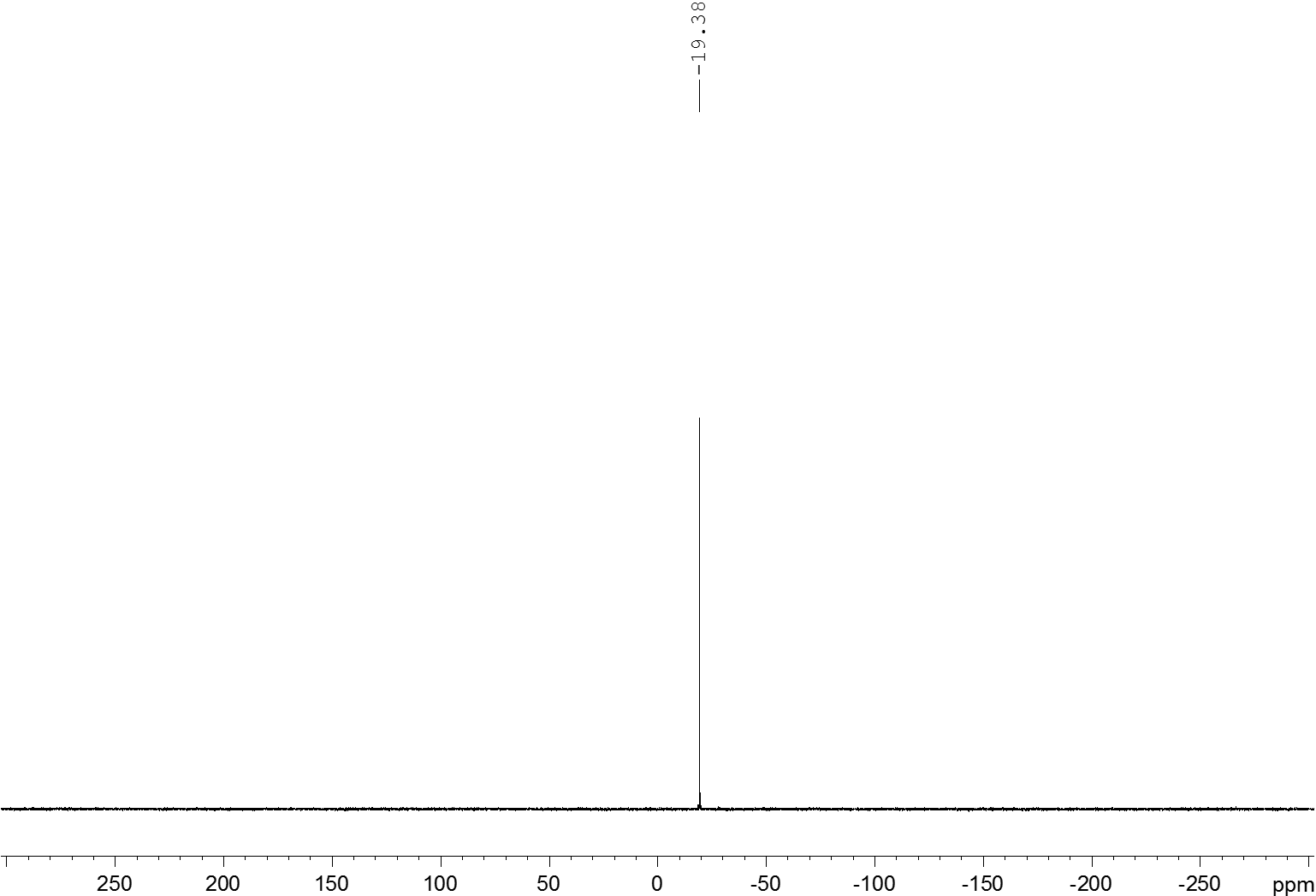
3. NMR and HRMS Spectra

**Figure S5.** The ^31^P {^1^H} NMR spectrum of **3** in CD_2_Cl_2_

**
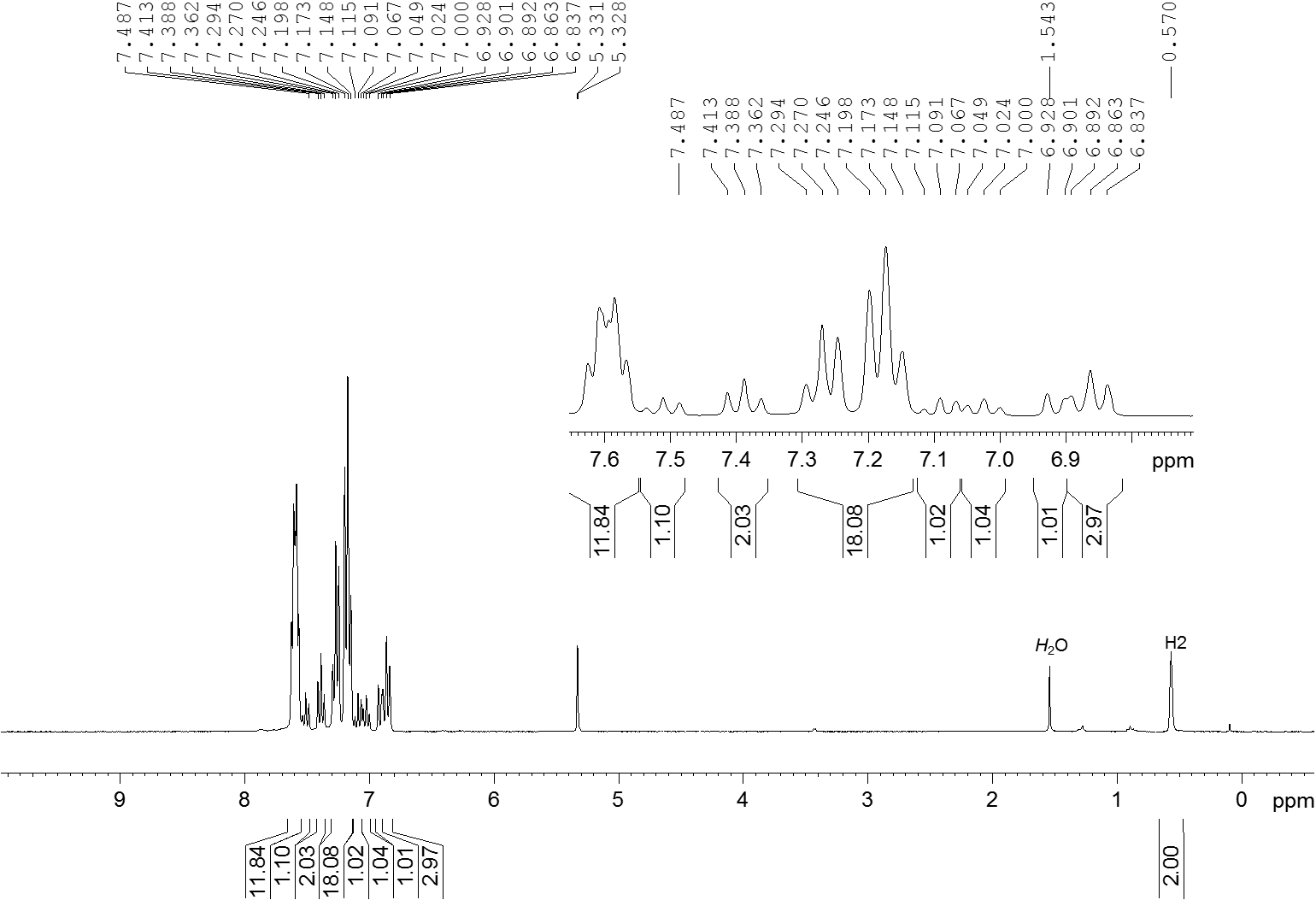
**

**Figure S6.** The ^1^H NMR spectrum of **3** in CD_2_Cl_2_

**
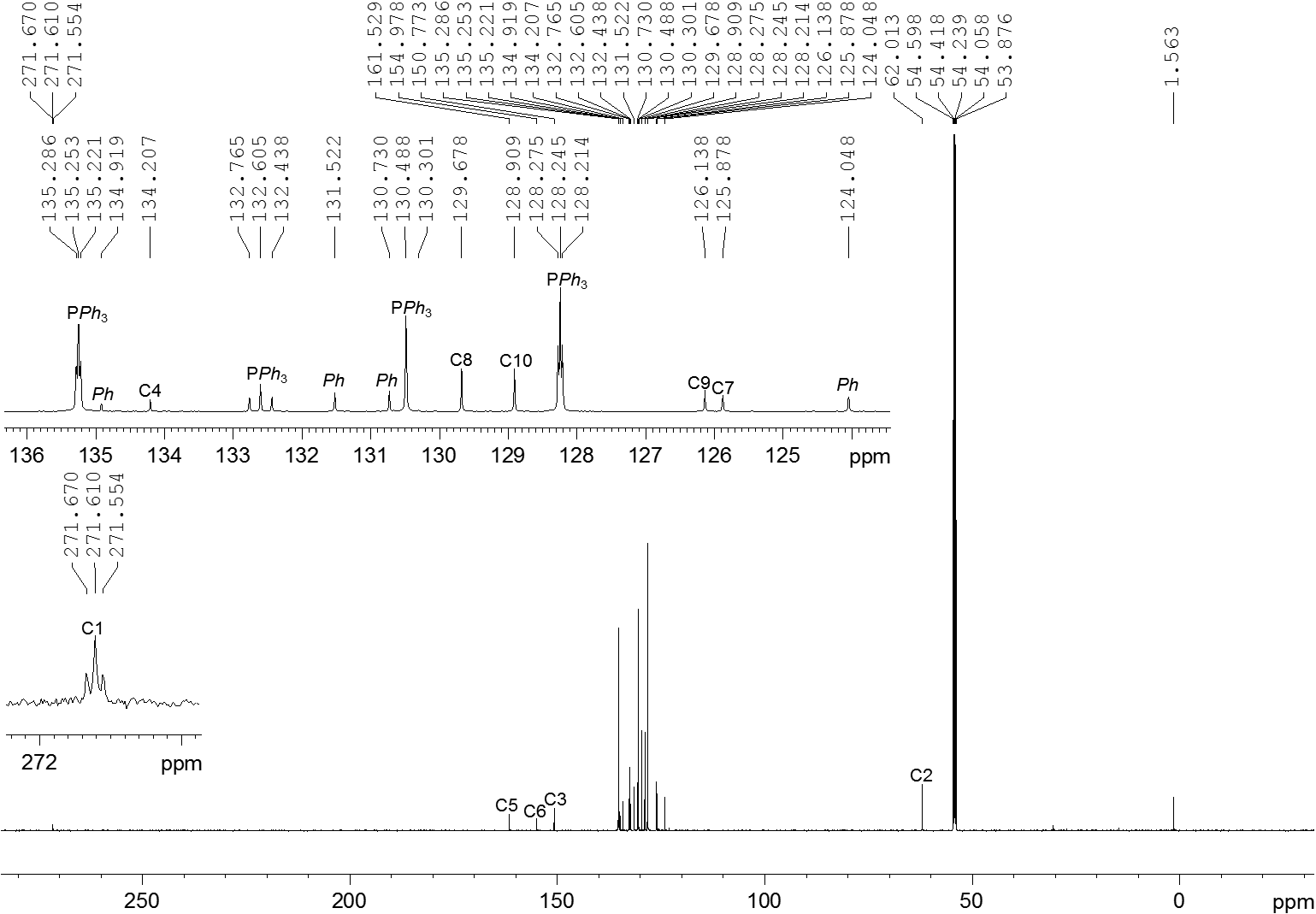
**


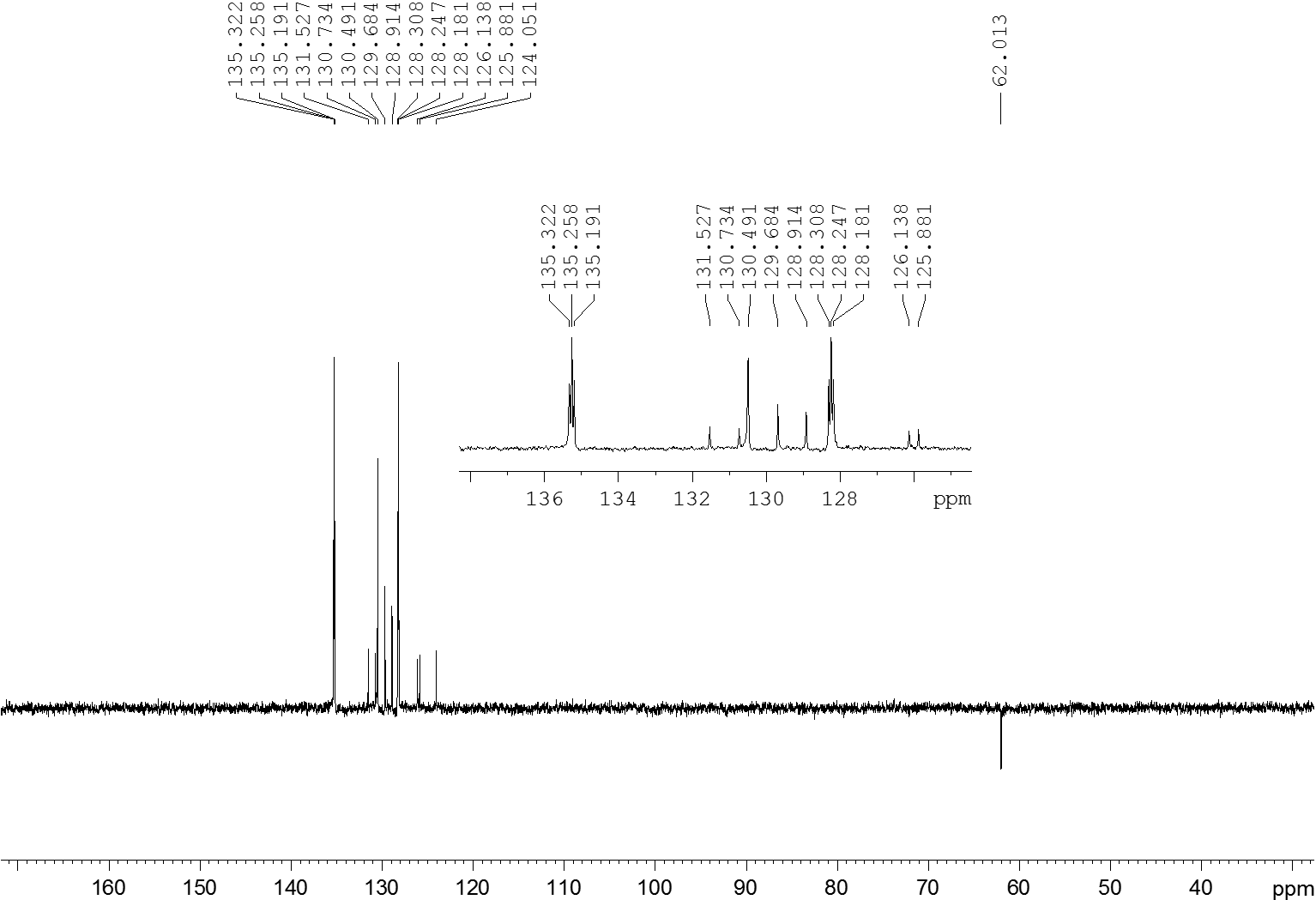
**Figure S7.** The ^13^C{^1^H} NMR spectrum of **3** in CD_2_Cl_2_

**Figure S8.** The ^13^C DEPT135 NMR spectrum of **3** in CD_2_Cl_2_

**
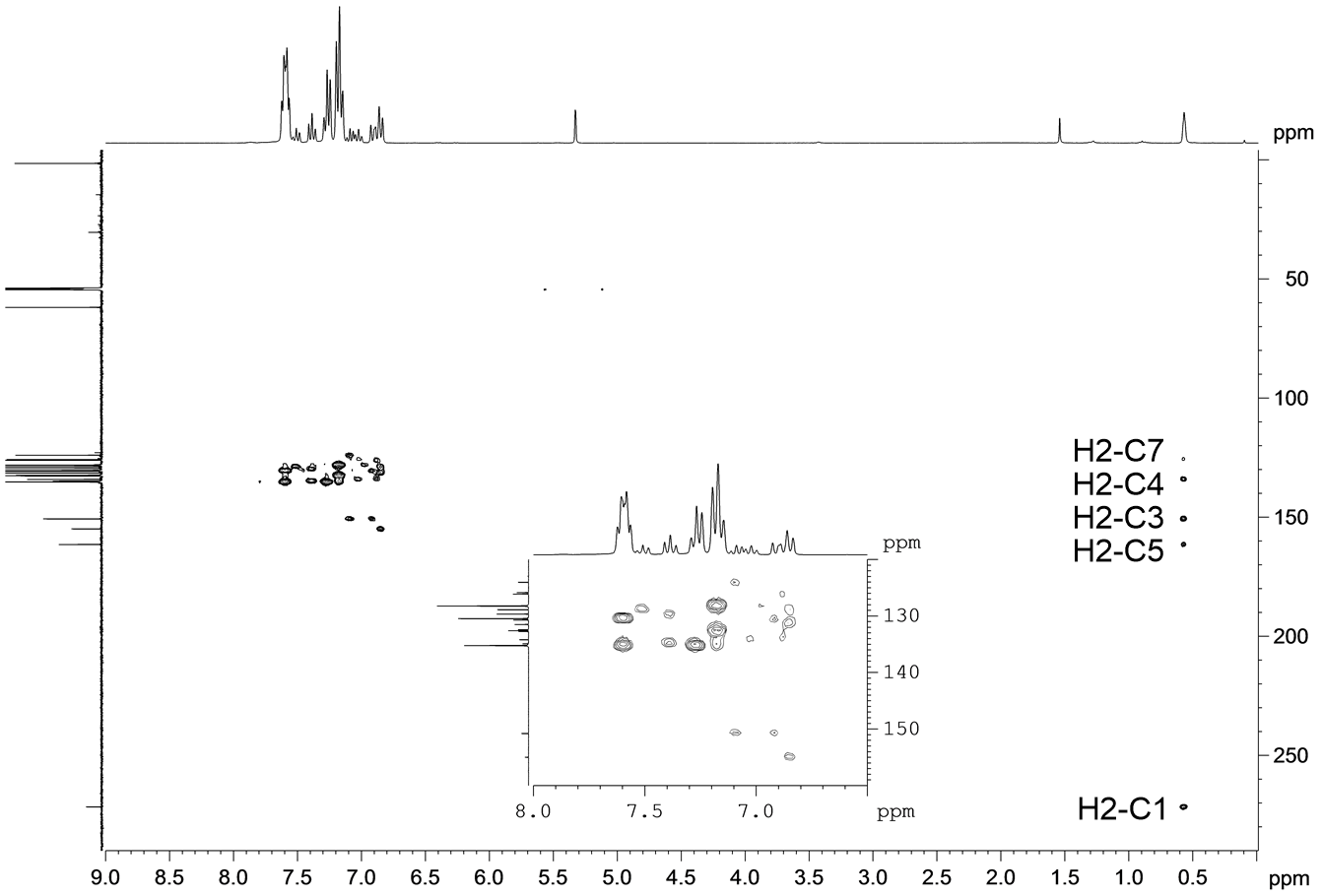
**

**
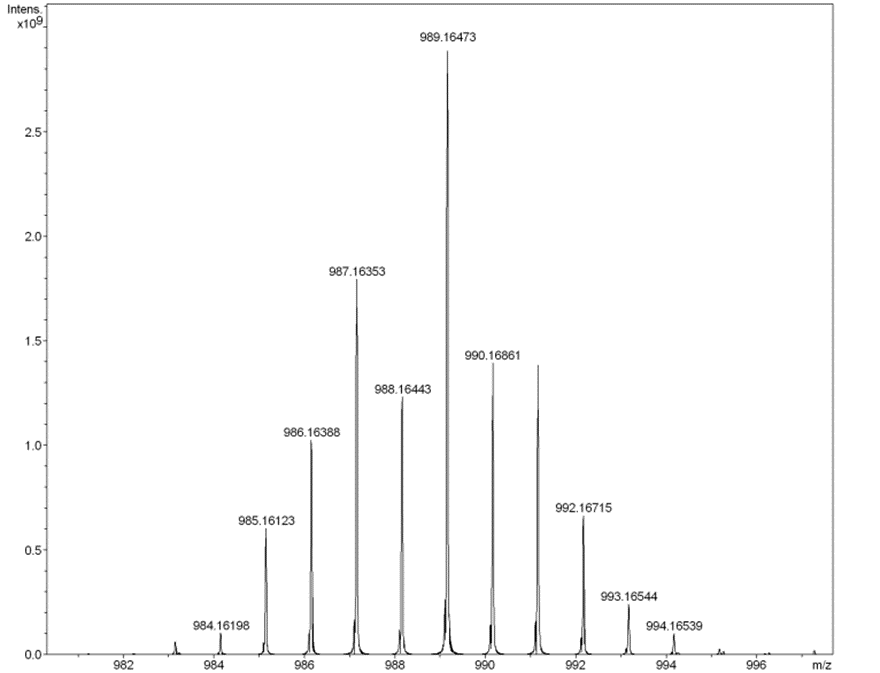
Figure S9.** The ^1^H-^13^C HMBC spectrum of **3** in CD_2_Cl_2_

**Figure S10.** The HRMS spectrum of [**3-Cl**]^+^ measured in dichloromethane.


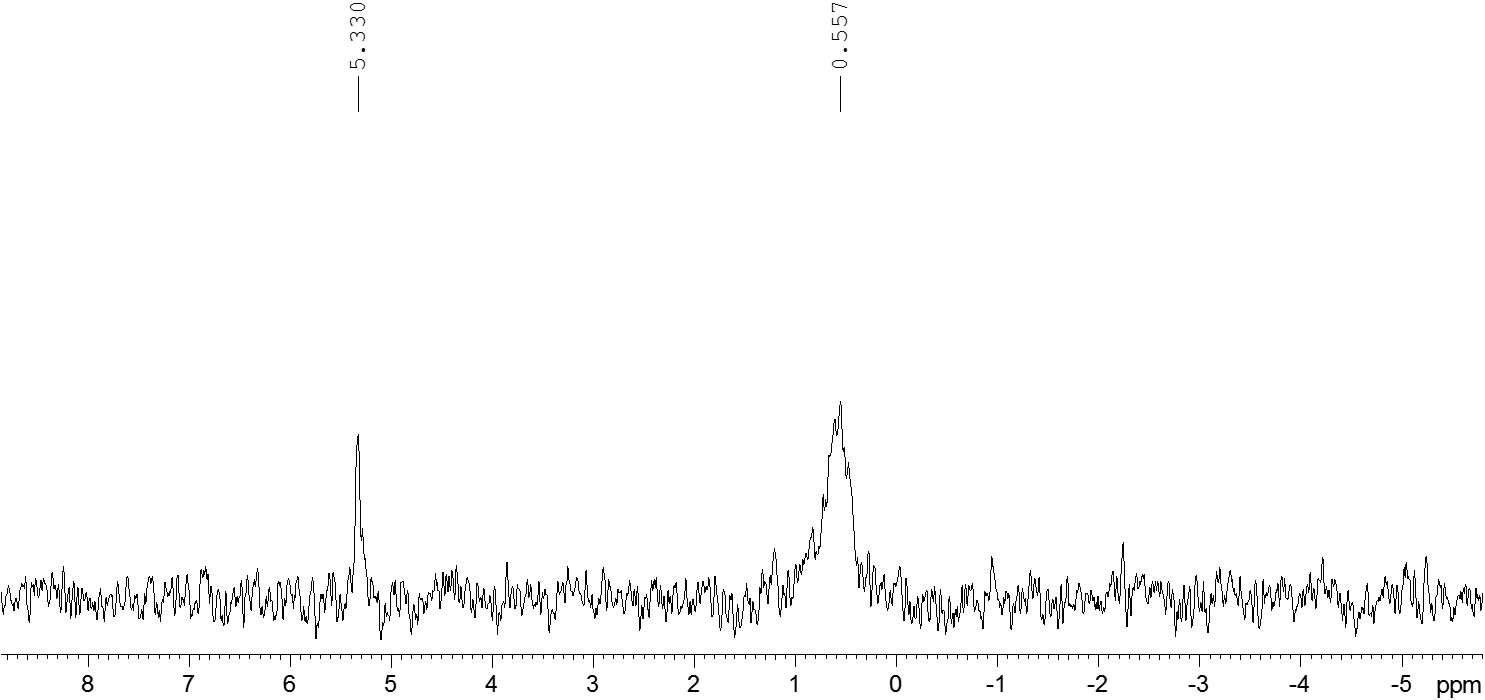

**Figure S11.** The ^2^D NMR spectrum of **3D** in CD_2_Cl_2_/CH_2_Cl_2_.

**
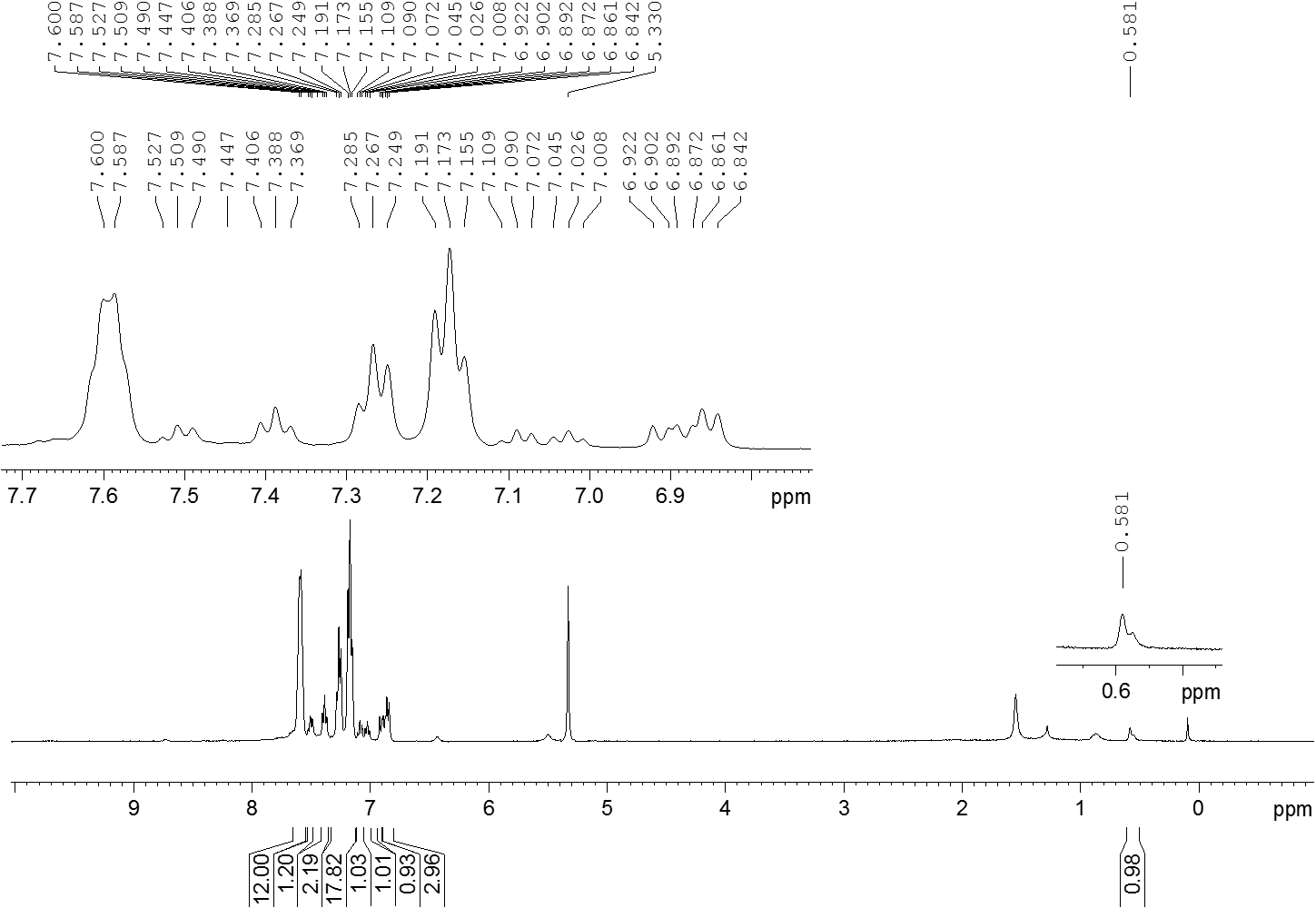
**

**Figure S12.** The ^1^H NMR spectrum of **3D** in CD_2_Cl_2_

**
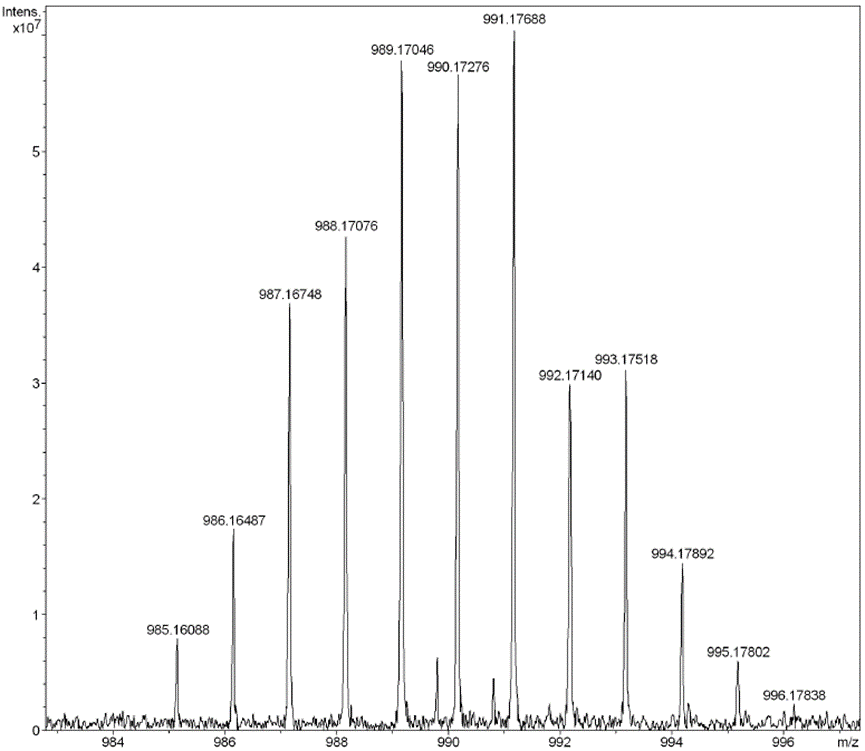
**

**
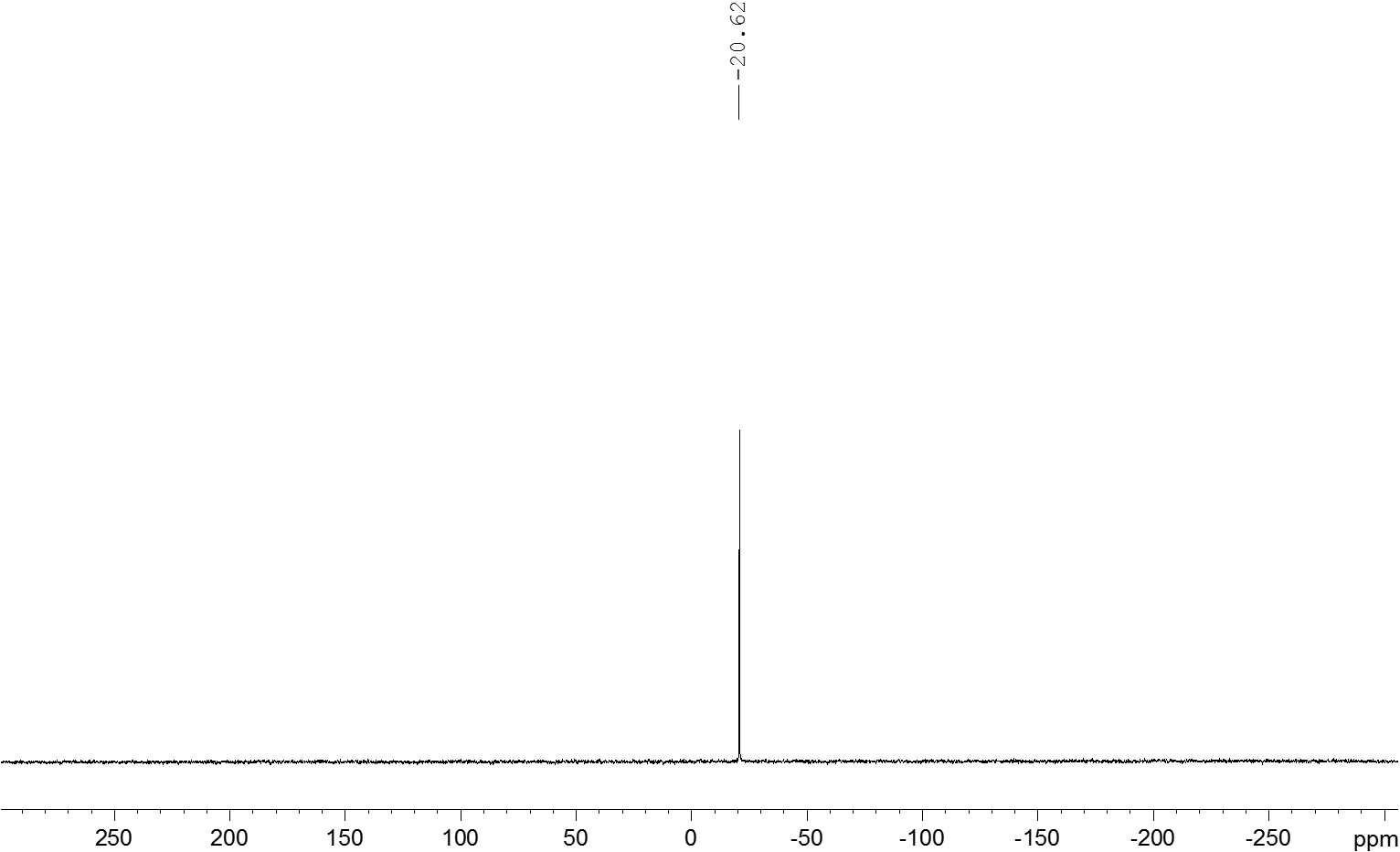
Figure S13.** The HRMS spectrum of [**3D-Cl**]^+^ measured in dichloromethane.

**Figure S14.** The ^31^P{^1^H} NMR spectrum of **10** in CD_2_Cl_2_

**
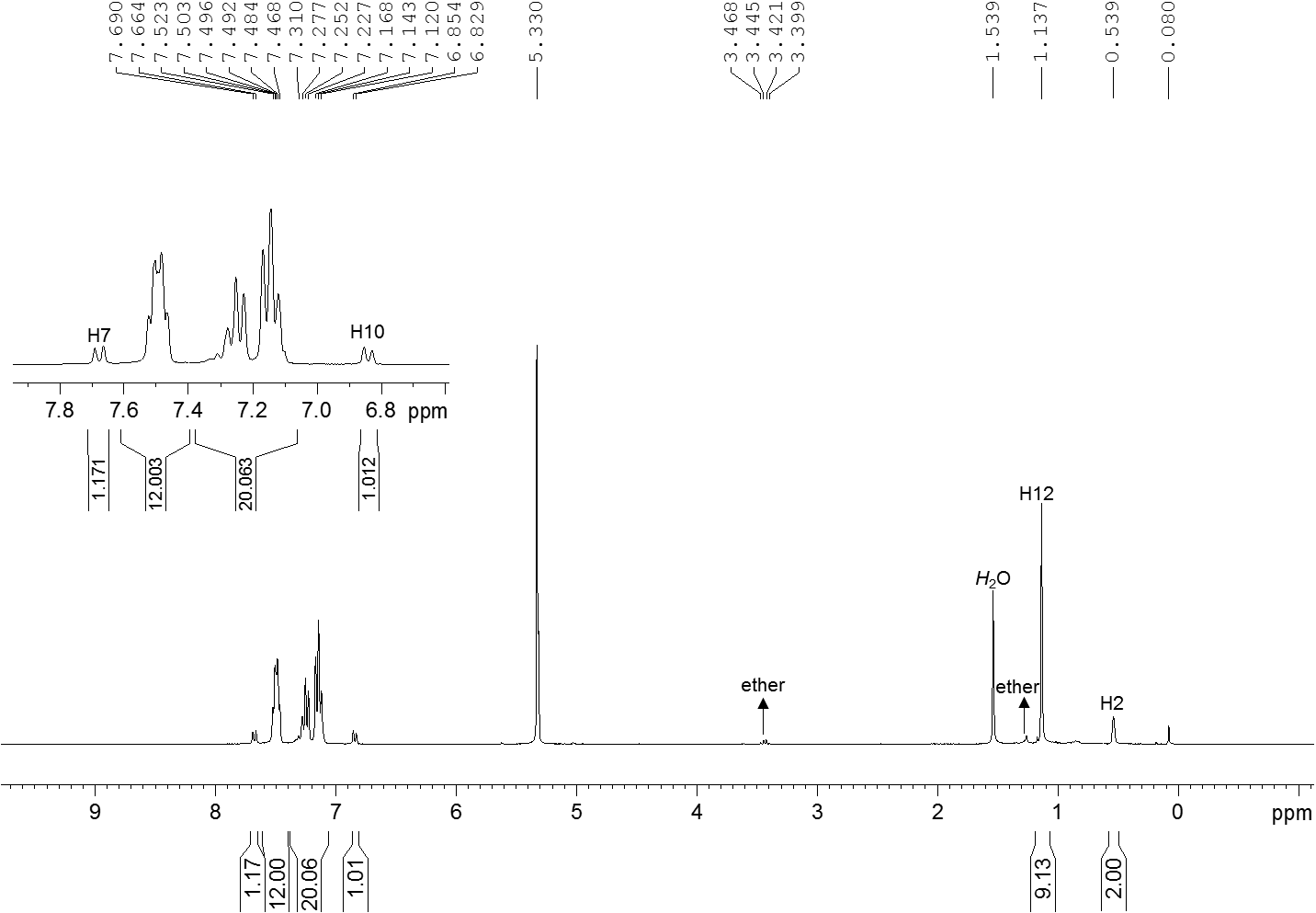
**

**
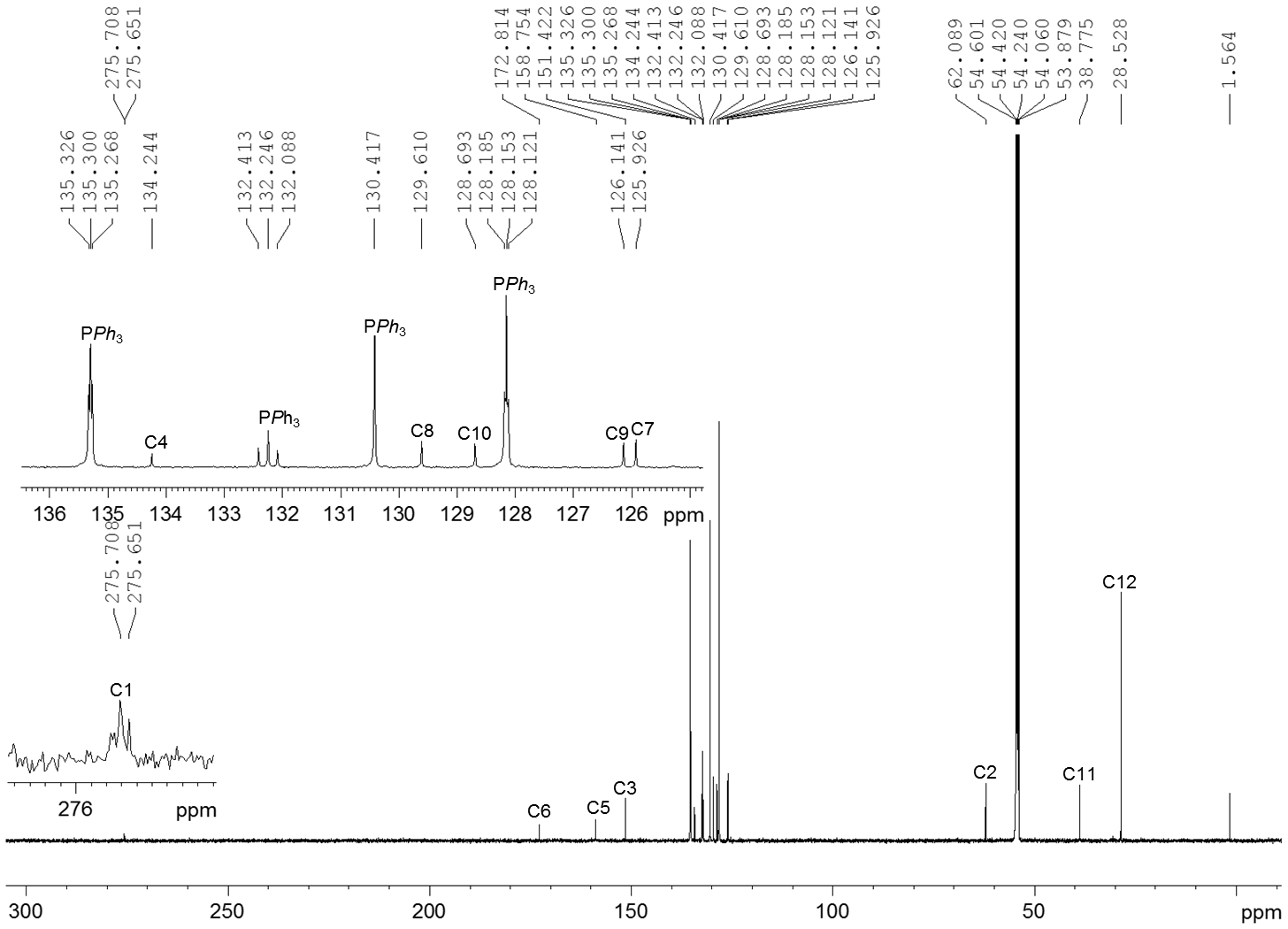
Figure S15.** The ^1^H NMR spectrum of **1**0 in CD_2_Cl_2_

**Figure S16. T**he ^13^C{^1^H} NMR spectrum of **10** in CD_2_Cl_2_

**
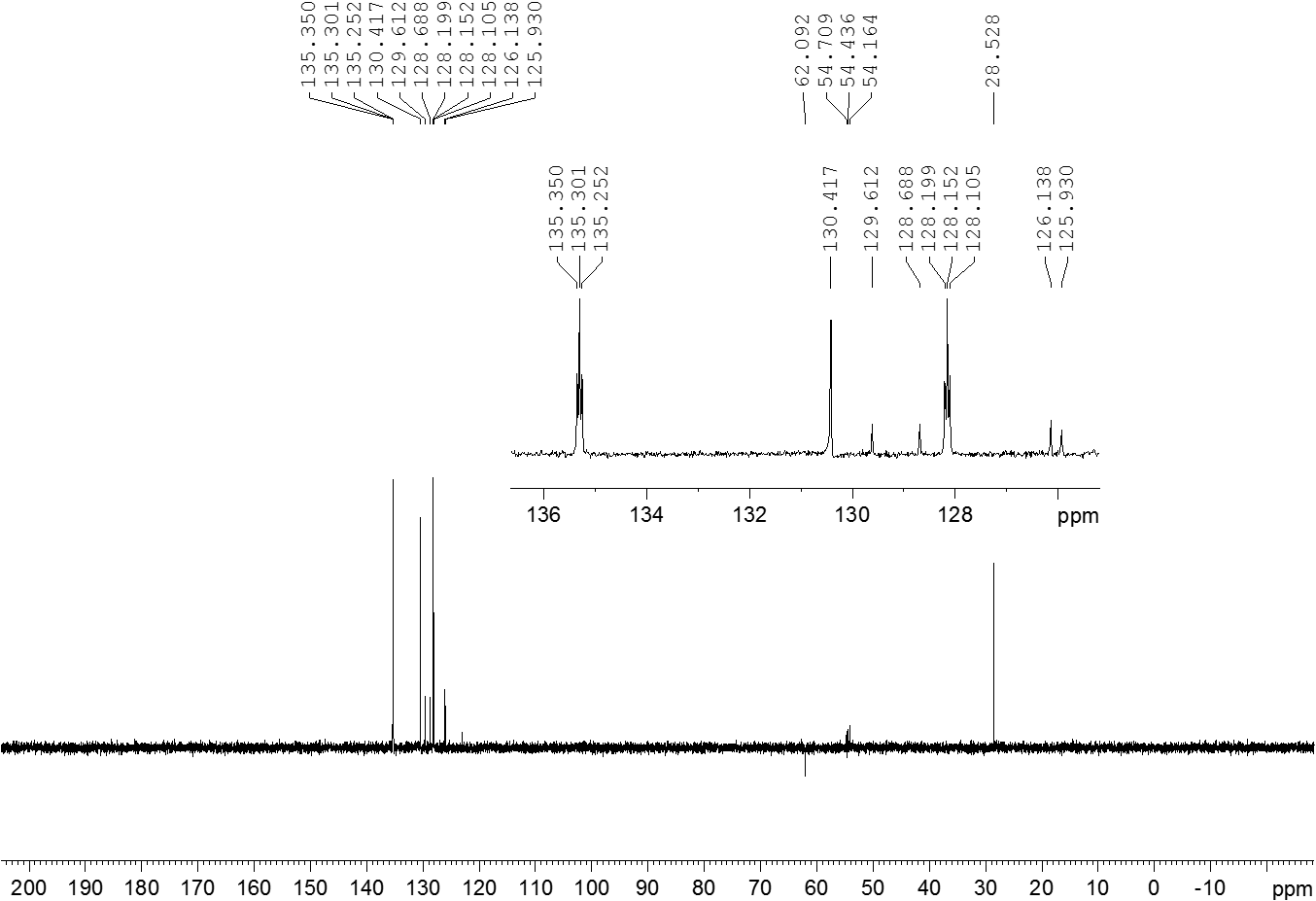
**

**
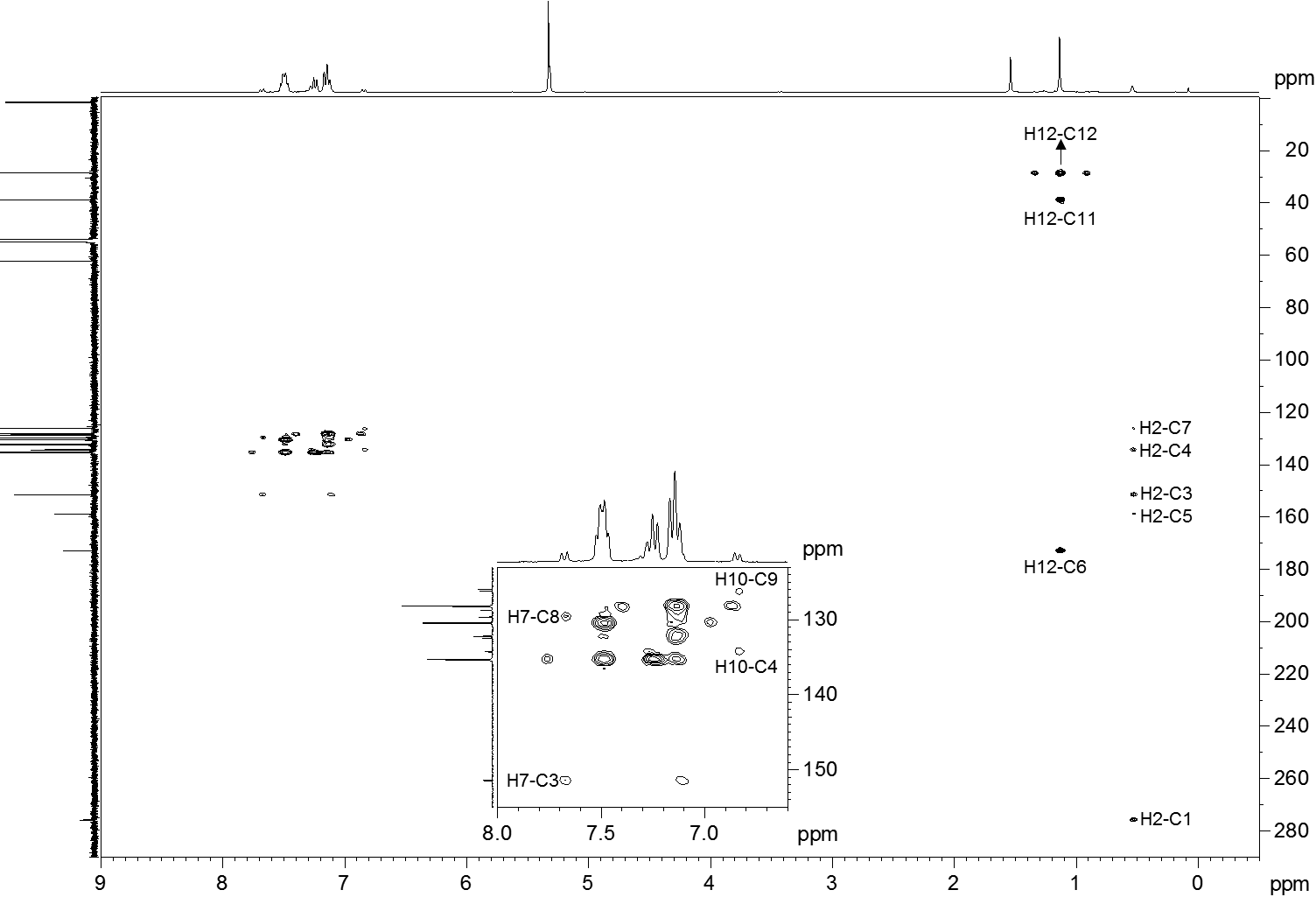
Figure S17.** The ^13^C DEPT135 NMR spectrum of **10** in CD_2_Cl_2_

**Figure S18.** The ^1^H-^13^C HMBC spectrum of **10** in CD_2_Cl_2_

**
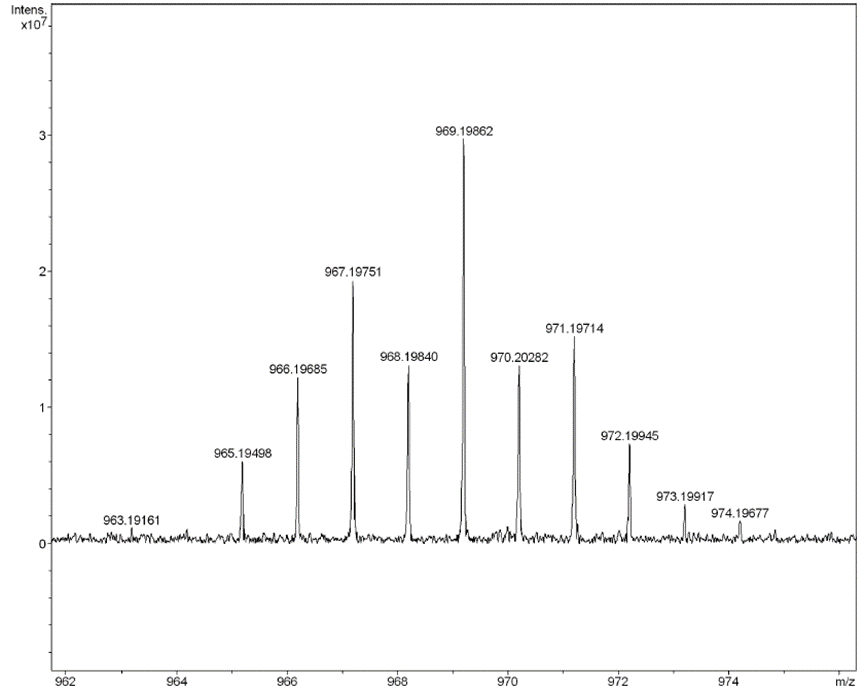
**

**Figure S19** The HRMS spectrum of [**10-Cl**]^+^ measured in dichloromethane.

**
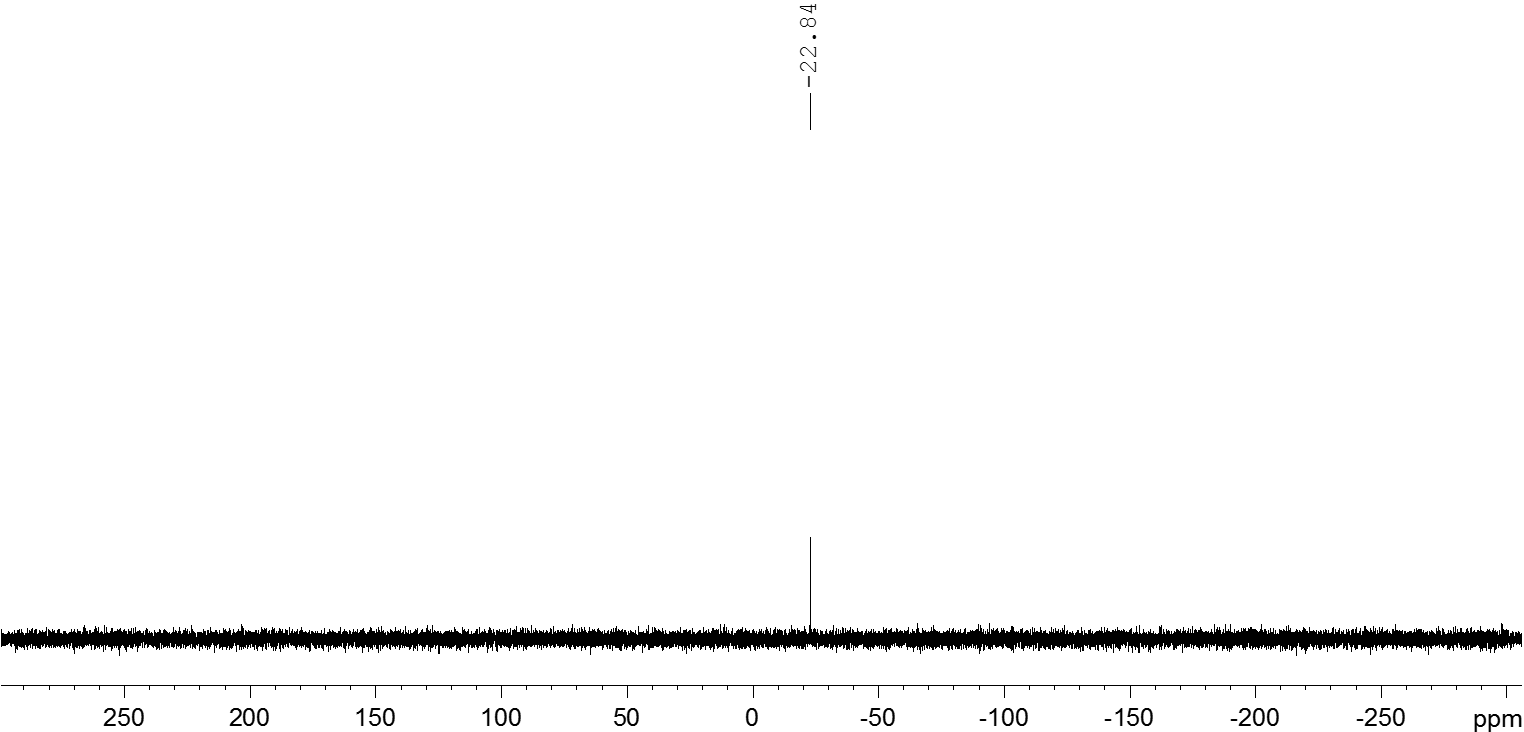
**

**Figure S20.** The ^31^P{^1^H} NMR spectrum of **12** in CD_2_Cl_2_

**
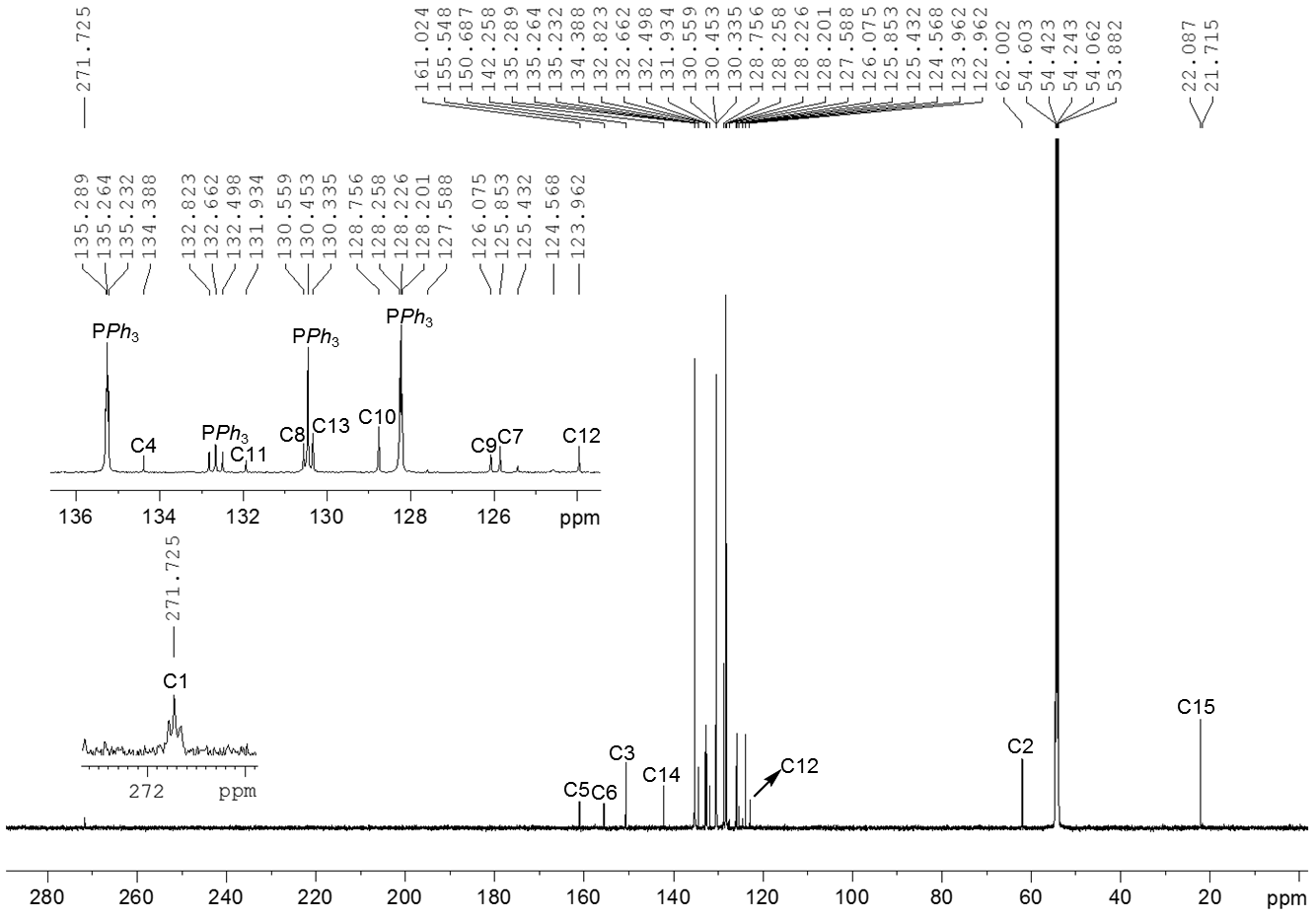

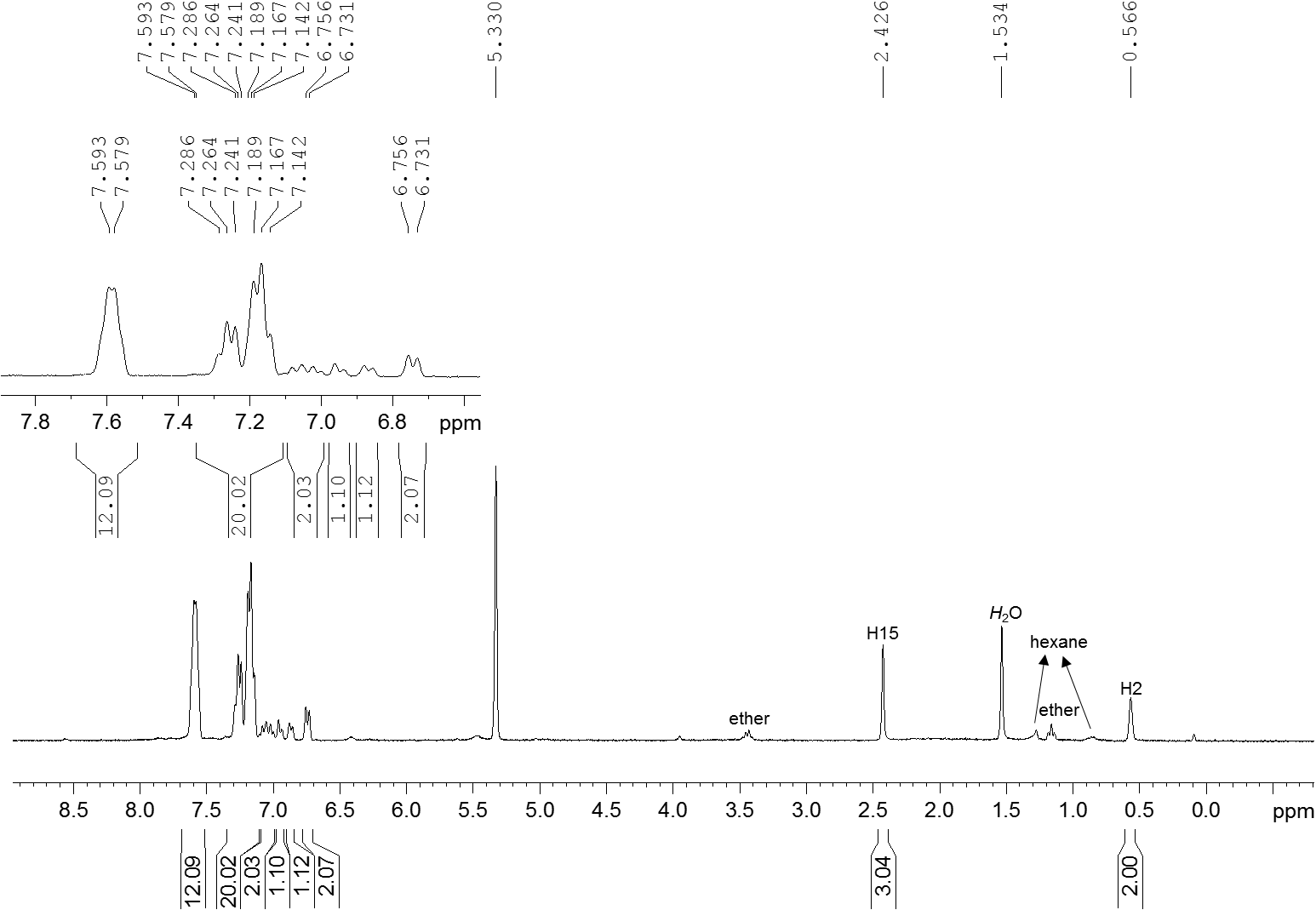
Figure S21.** The ^1^H NMR spectrum of **12** in CD_2_Cl_2_

**Figure S22.** The ^13^C{^1^H} NMR spectrum of **12** in CD_2_Cl_2_

**
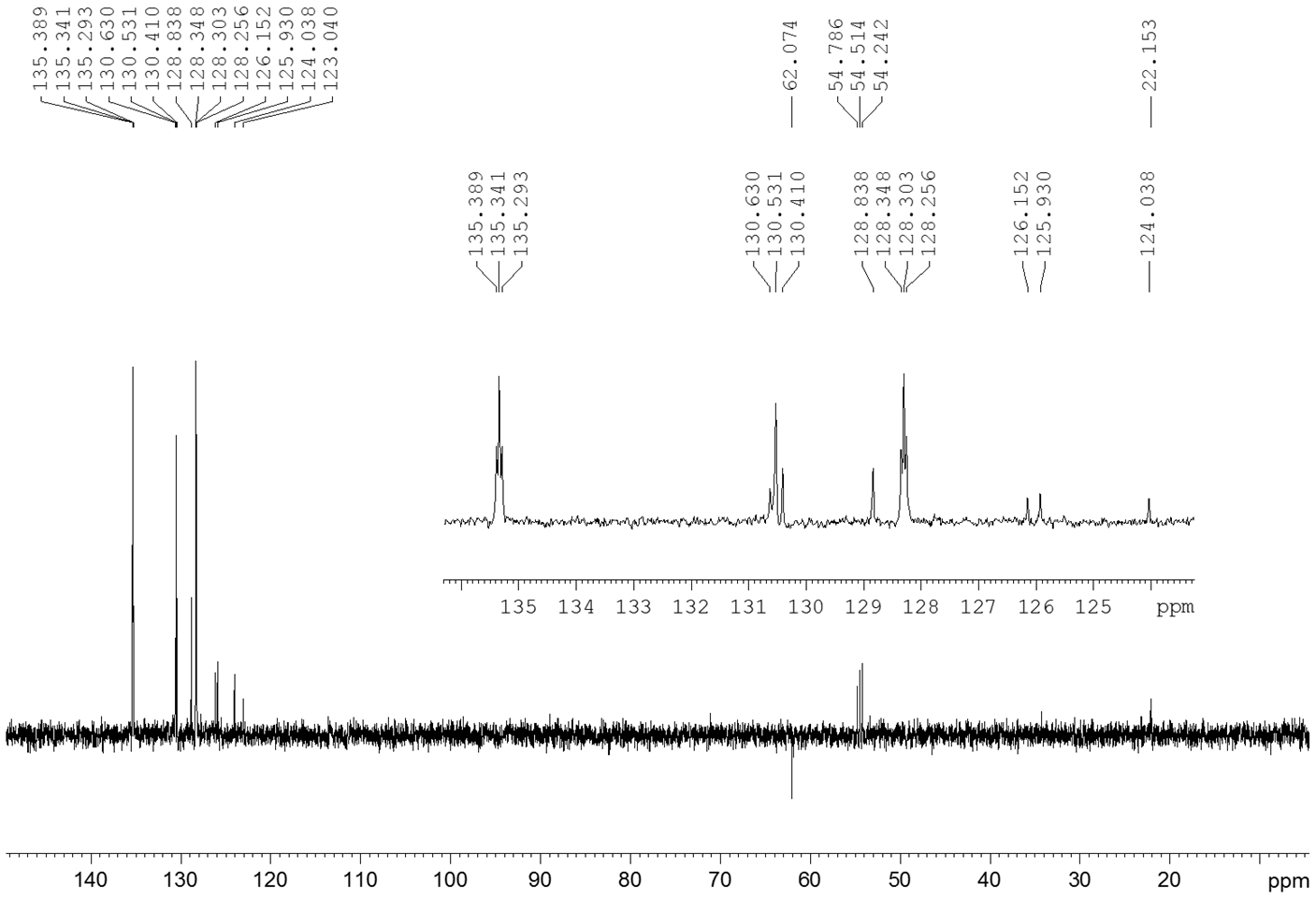
**

**
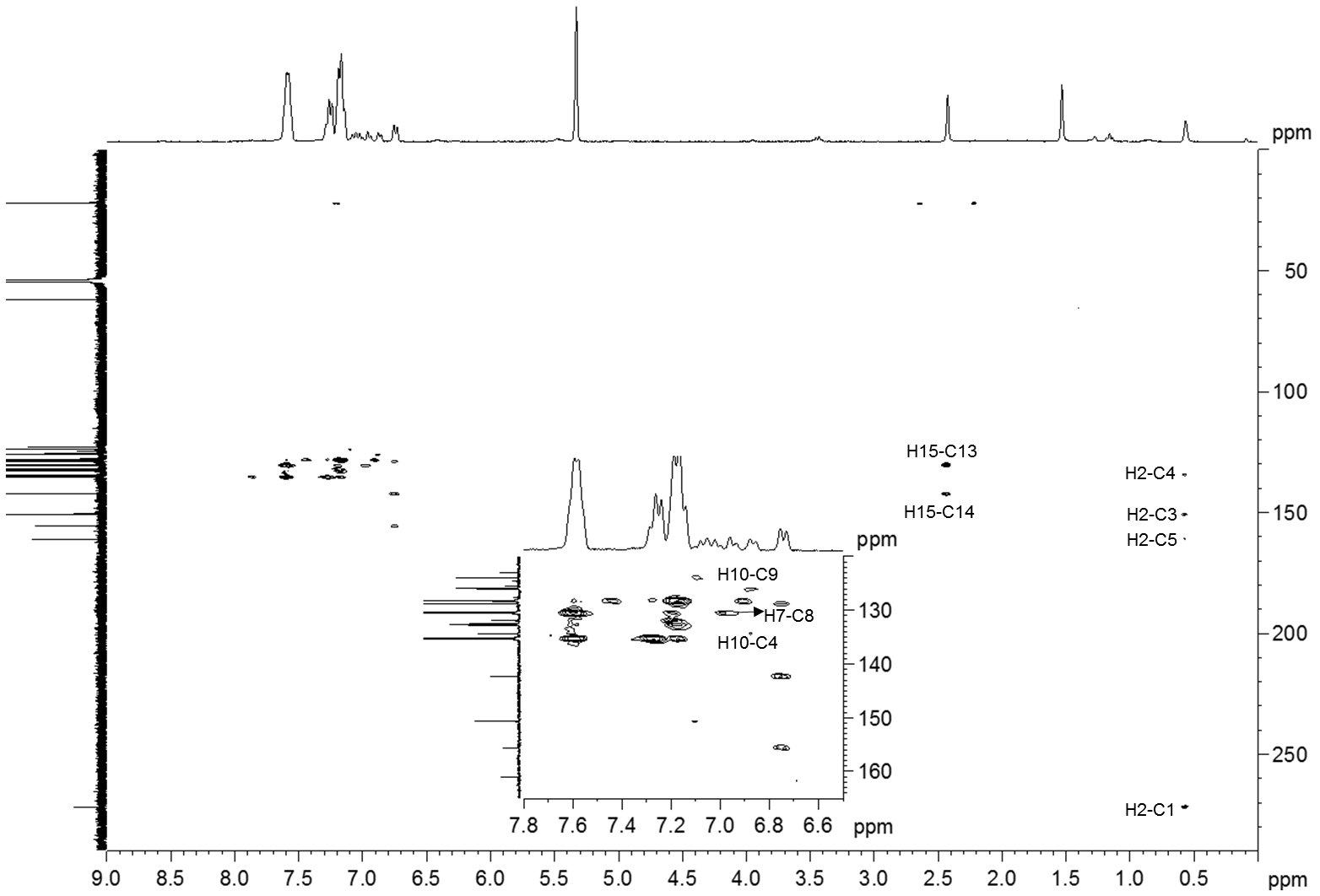
Figure S23.** The ^13^C DEPT135 NMR spectrum of **12** in CD_2_Cl_2_

**Figure S24. T**he ^1^H-^13^C HMBC spectrum of **12** in CD_2_Cl_2_

**
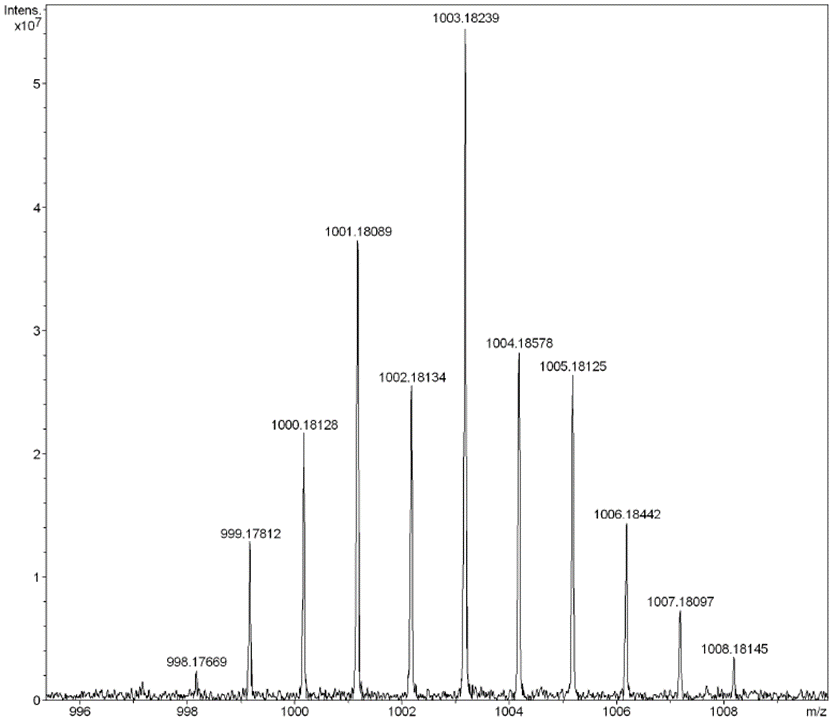
**

**
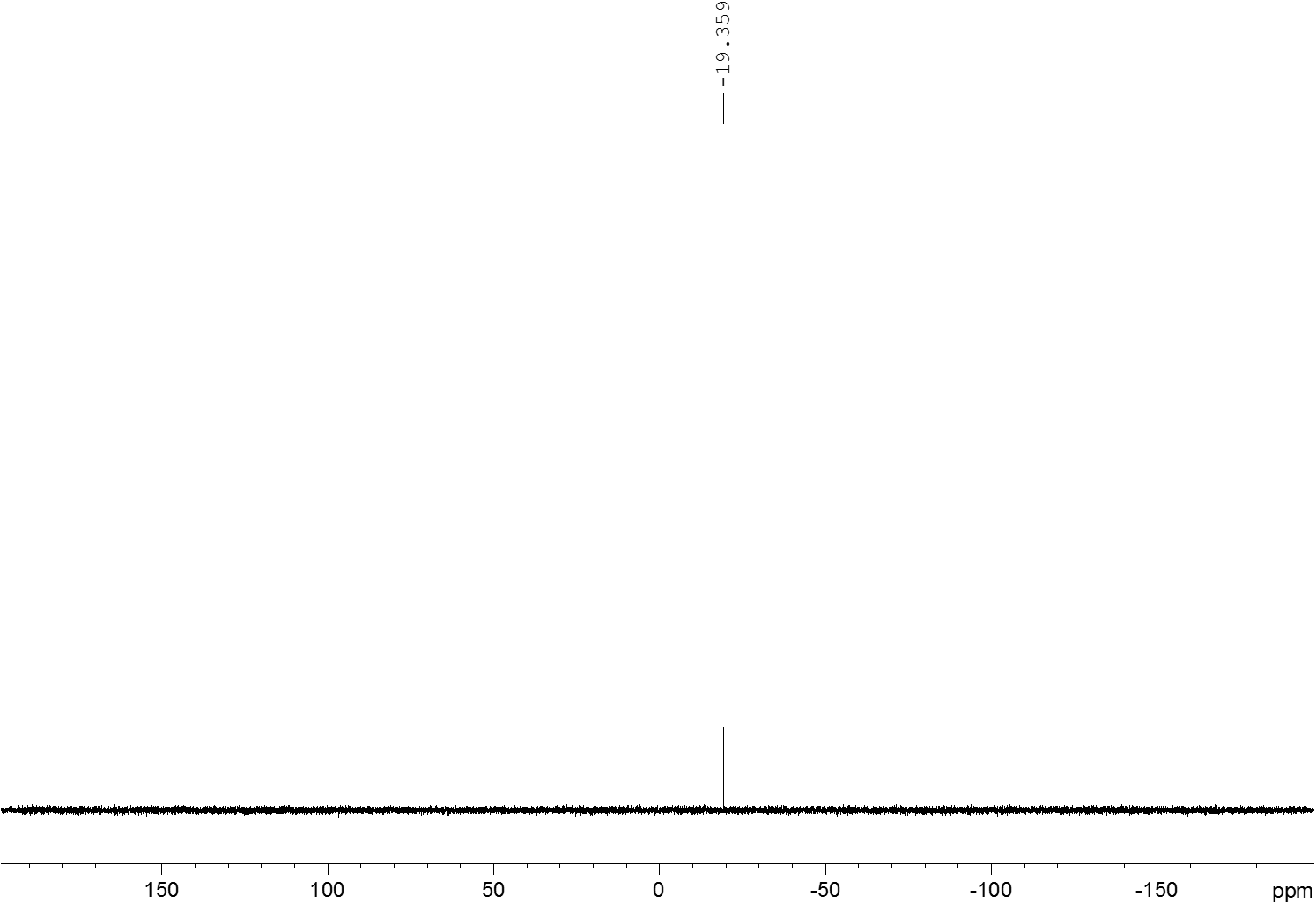
Figure S25.** The HRMS spectrum of [**12-Cl**]^+^ measured in dichloromethane.

**Figure S26.** The ^31^P{^1^H} NMR spectrum of **14** in CD_2_Cl_2_

**
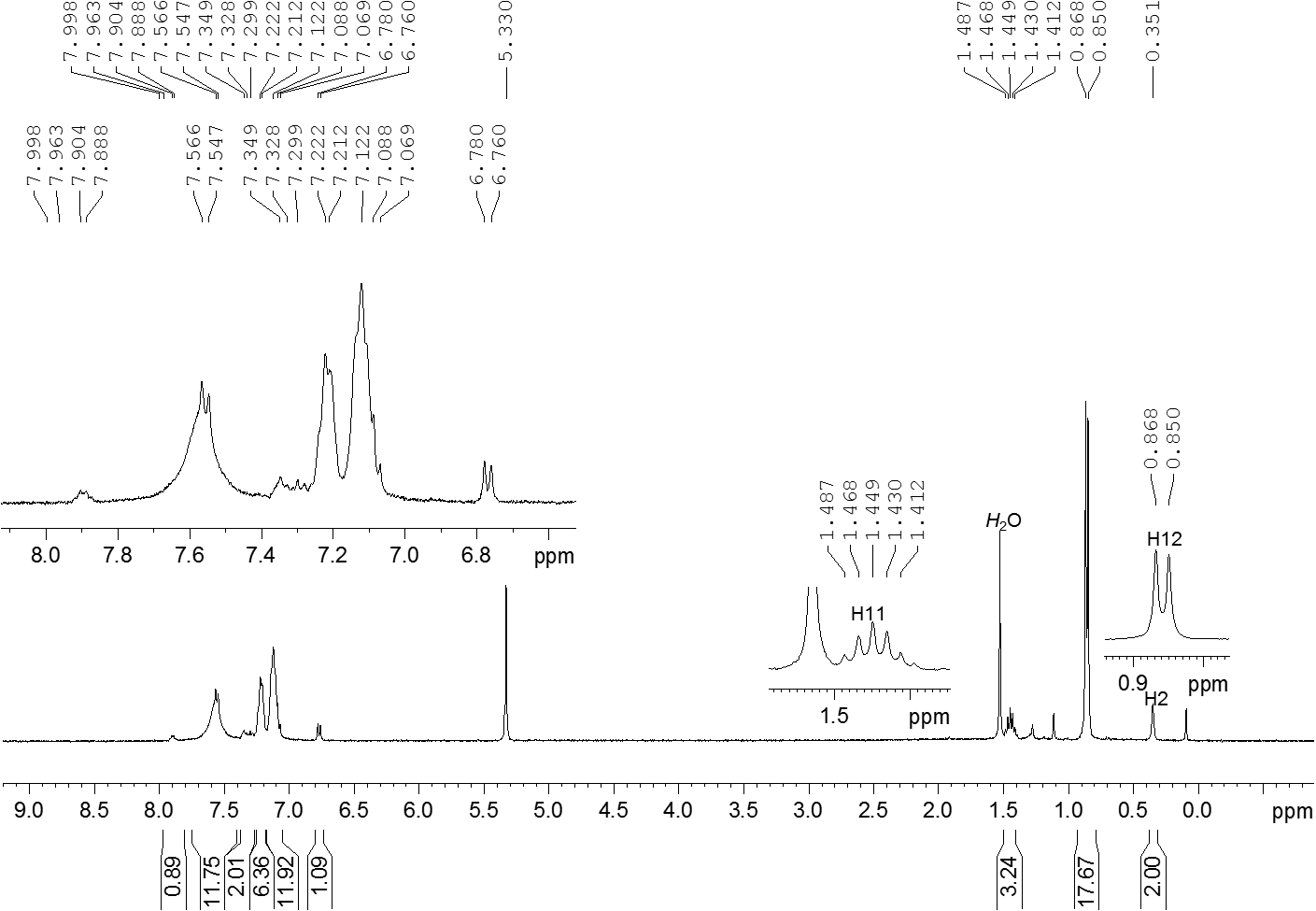
**

**
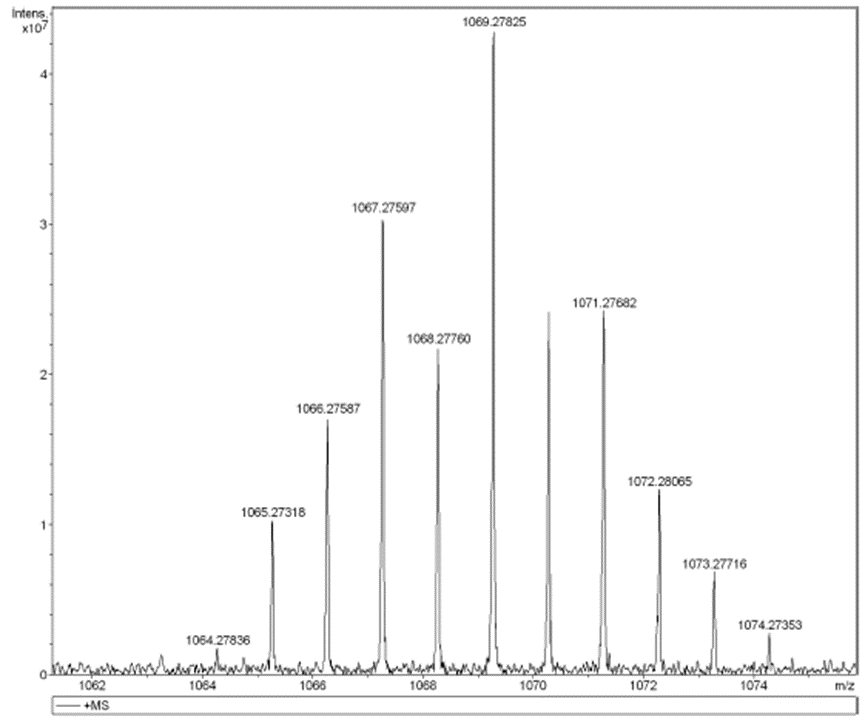
Figure S27.** The ^1^H NMR spectrum of **14** in CD_2_Cl_2_

**Figure S28.** The HRMS spectrum of [**14-Cl**]^+^ measured in dichloromethane.

4. Computational Details

All the optimizations were performed with the Gaussian 16 rev. C01 software package [8]. All of these structures evaluated were optimized at the PBE/def2tzvp level of density functional theory (DFT) [9-12]. Frequency analyses have been performed to validate the stationary points as intermediate with zero imaginary frequency. Nucleus-independent chemical shift (NICS) values were calculated at the PBE-GIAO/x2c-TZVPall level [13-16] based on the optimized structures.

5. The Calculated Cartesian Coordinates with Electronic Energies

Complex **3**

E = -4157.71683778 a.u.

Os 2.61237700 4.73211200 0.36983700

Cl 0.39469400 5.46370900 0.21095100

Cl 3.49863900 7.09702500 0.66432800

P 3.03402300 5.11573300 -1.99353200

P 2.56340300 4.70562500 2.80568000

Cl 4.78612300 3.67638000 0.49841100

C 1.78857400 3.01605000 0.14160900

C 1.75945800 6.13960000 -2.85863500

C 4.57795900 5.98422700 -2.51755200

C 0.30816600 2.83796900 0.03806400

C 3.90309400 5.56990300 3.73621000

C -0.51667000 3.91742500 0.02880100

C 2.59634000 3.01382100 3.53547100

C 1.52004300 2.13390100 3.32861100

H 0.64893100 2.45917200 2.75787500

C 0.00000000 1.39969200 0.00000000

C 1.06348700 5.51035000 3.52745500

C 1.13944000 5.77456900 -4.06228800

H 1.37856600 4.82315800 -4.53808800

C 2.38521700 1.62588000 0.05071700

H 3.03200100 1.55650000 -0.84056000

H 3.06254200 1.43809900 0.89925600

C 5.18011000 5.74939000 3.18739900

H 5.38148300 5.41021700 2.17249000

C 1.21742600 0.68367700 0.00000000

C 3.11306600 3.54502500 -2.95152500

C 5.67783300 6.13439500 -1.66364500

H 5.62331700 5.75549100 -0.64555400

C 1.45301000 7.38622500 -2.28464700

H 1.94586200 7.68511400 -1.35491800

C 1.21704100 -0.70976300 -0.01178100

H 2.15979100 -1.26144200 -0.01183000

C 6.83794200 6.76412100 -2.12208300

H 7.68630300 6.87858900 -1.44472200

C 0.82786800 6.84662400 3.15539200

H 1.51625300 7.34036900 2.46299300

C 4.34471200 3.03306100 -3.38503300

H 5.25949300 3.59926100 -3.20924500

C 1.95067200 2.78456900 -3.17001600

H 0.98573400 3.14722900 -2.81152100

C 4.65603000 6.47897400 -3.83081500

H 3.80414100 6.38295000 -4.50574200

C 3.71655700 2.55292700 4.24262800

H 4.56787100 3.21428300 4.40327400

C 0.53974200 8.24007600 -2.90205800

H 0.31527800 9.20627000 -2.44600300

C 5.81684300 7.10641700 -4.28259300

H 5.86101000 7.48814800 -5.30443000

C 4.40928000 1.79728700 -4.03489600

H 5.37765500 1.41190900 -4.35935800

C 0.18420100 4.88846700 4.42424900

H 0.36108100 3.86150600 4.74458600

C 3.65279900 6.00673200 5.04780300

H 2.66310100 5.88214600 5.48921400

C -1.14943700 6.90763800 4.55475400

H -2.00779300 7.45062800 4.95526300

C 6.91228200 7.25118400 -3.42740100

H 7.81929100 7.74749000 -3.77849300

C -0.08451300 7.86491600 -4.09510100

H -0.80194500 8.53388100 -4.57423200

C 3.75299300 1.24721400 4.73908700

H 4.63645900 0.90419200 5.28078800

C -0.91908000 5.58397400 4.93079200

H -1.59432400 5.08571300 5.62929700

C -0.26867700 7.53760300 3.66933500

H -0.43523200 8.57591600 3.37588000

C 1.55323600 0.83498800 3.83602900

H 0.70614000 0.16846600 3.66365000

C 2.01614400 1.55815900 -3.83127800

H 1.10235800 0.98398600 -3.99545000

C 0.21923500 6.63219600 -4.67324300

H -0.25532800 6.33276800 -5.60991500

C 4.66335700 6.61089700 5.79692600

H 4.45207200 6.94873400 6.81331500

C -1.22109500 0.70822000 0.01459500

H -2.16989300 1.24426800 0.03992000

C 2.67165400 0.38733100 4.54439700

H 2.70139400 -0.63121400 4.93583600

C 3.24746100 1.06061000 -4.26756300

H 3.29950600 0.09814400 -4.77976300

C 6.18796000 6.35191200 3.94351600

H 7.17688800 6.48954900 3.50220500

C -0.00207900 -1.39447800 -0.01225000

H -0.01299600 -2.48613900 -0.02149800

C 5.93468300 6.78567200 5.24576600

H 6.72422100 7.26279300 5.82988200

C -1.21129300 -0.68734700 0.00943600

H -2.15703800 -1.23241700 0.02303800

C -4.72007600 4.48631800 -0.47384600

H -5.79298300 4.63471300 -0.60696500

C -4.00691200 3.67961000 -1.36561000

H -4.51922200 3.20396100 -2.20389000

C -2.63710900 3.48878000 -1.19873300

H -2.07944700 2.87337100 -1.90613300

C -1.95387800 4.10130600 -0.12902800

C -4.05071700 5.10824400 0.58328400

H -4.60032300 5.74115600 1.28226600

C -2.67960200 4.92607000 0.75414500

H -2.16139200 5.41054600 1.58379000

Complex **3M**

E = -2312.20758275 a.u.

Os -0.51455400 -0.00163500 -0.17786400

P -1.00098000 2.27063500 0.10964000

P -0.99159500 -2.27590900 0.10952200

Cl 1.57951500 0.00277200 0.96021000

Cl -2.44334300 -0.00560900 -1.59660900

Cl -1.93727200 -0.00463700 1.92190100

C 0.62360200 0.00074200 -1.71384100

C 2.07485100 0.00385100 -1.61592700

C 2.70490800 0.00517900 -0.42061000

H 2.69728200 0.00518600 -2.51684700

H 0.23667900 -0.00015900 -2.74723100

C 4.14562300 0.00827100 -0.06903600

H 4.75268100 0.00955000 -0.98390700

H 4.41152200 0.89476400 0.52805200

H 4.41532100 -0.87703800 0.52810300

H -0.63486500 2.90597000 1.32610300

H -0.52830200 3.25572700 -0.80673200

H -2.38739600 2.54667700 0.06721200

H -0.62283600 -2.90984000 1.32592000

H -0.51491500 -3.25898700 -0.80694000

H -2.37687000 -2.55762900 0.06712600

Complex **3H**

E = -2312.18785783 a.u.

Os -0.01802100 -0.05453300 -0.15410100

P -2.34692100 -0.10564800 0.12292400

P 2.15549200 -0.90220000 0.11472600

Cl 0.35124800 1.93020200 1.12877300

Cl -0.35723200 -1.89077700 -1.65055900

Cl -0.28318000 -1.55183600 1.87114500

C 0.16648300 1.10805900 -1.62514000

H 0.05313500 0.77798100 -2.67527200

C 0.44486400 2.59518200 -1.53766800

H 1.29712400 2.84586400 -2.19554400

H -0.42054400 3.12401800 -1.98461200

C 0.70600200 3.13862700 -0.16757100

C 1.15022400 4.33708100 0.20136600

H 1.30148100 4.60588100 1.24647500

H 1.37247600 5.09160000 -0.55510200

H -2.90672100 0.34915200 1.34656500

H -3.22139200 0.56404800 -0.78221600

H -2.88266900 -1.41265100 0.05048100

H 2.84477600 -0.65620100 1.33217200

H 3.20113400 -0.58381200 -0.80004600

H 2.20920600 -2.31441600 0.05679900

# Supplemental References

1 Hoffman PRand Caulton KG. Solution structure and dynamics of five-coordinate d^6^ complexes. *J Am Chem Soc* 1975; **97**: 4221-8.

2 Schmittel M and Strittmatter M. Cyclization of carbonyl substituted enyne-allenes: C^2^-C^6^-cyclization induced by heat or by addition of samarium(II) iodide, samarium(III) chloride, or boron trifluoride. *Tetrahedron* 1998; **54**: 13751-60.

3 Byers PM, Rashid JI and Mohamed RK *et al.* Polyaromatic ribbon/benzofuran fusion via consecutive endo cyclizations of enediynes. *Org Lett* 2012; **14**: 6032-5.

4 Bell ML, Chiechi RC and Johnson CA *et al.* A versatile synthetic route to dehydrobenzoannulenes via in situ generation of reactive alkynes. *Tetrahedron* 2001; **57**: 3507-20.

5 Dolomanov OV, Bourhis LJ and Gildea RJ *et al.* OLEX2: a complete structure solution, refinement and analysis program. *J Appl Cryst* 2009; **42**: 339-41.

6 Sheldrick G. SHELXT - Integrated space-group and crystal-structure determination. *Acta Cryst Sect A* 2015; **71**: 3-8.

7 Sheldrick G. Crystal structure refinement with SHELXL. *Acta Cryst. Sect C* 2015; **71**: 3-8.

8 Gaussian 16, Revision C.01, Frisch MJ, Trucks GW, Schlegel HB, Scuseria GE, Robb MA, Cheeseman JR, Scalmani G, Barone V, Petersson GA, Nakatsuji H, Li X, Caricato M, Marenich AV, Bloino J, Janesko BG, Gomperts R, Mennucci B, Hratchian HP, Ortiz JV, Izmaylov AF, Sonnenberg JL, Williams-Young D, Ding F, Lipparini F, Egidi F, Goings J, Peng B, Petrone A, Henderson T, Ranasinghe D, Zakrzewski VG, Gao J, Rega N, Zheng G, Liang W, Hada M, Ehara M, Toyota K, Fukuda R, Hasegawa J, Ishida M, Nakajima T, Honda Y, Kitao O, Nakai H, Vreven T, Throssell K, Montgomery JA, Jr., Peralta JE, Ogliaro F, Bearpark MJ, Heyd JJ, Brothers EN, Kudin KN, Staroverov VN, Keith TA, Kobayashi R, Normand J, Raghavachari K, Rendell AP, Burant JC, Iyengar SS, Tomasi J, Cossi M, Millam JM, Klene M, Adamo C, Cammi R, Ochterski JW, Martin RL, Morokuma K, Farkas O, Foresman JB and Fox DJ. Gaussian, Inc., Wallingford CT, 2019.

9 Perdew JP, Burke K and Ernzerhof M. Generalized gradient approximation made simple. *Phys Rev Lett* 1996; **77:** 3865-8

10 Ernzerhof M and Scuseria GE. Assessment of the Perdew–Burke–Ernzerhof exchange-correlation functional. *J Chem Phys* 1999; **110**: 5029-36.

11 Perdew JP, Burke K and Ernzerhof M. Generalized gradient approximation made simple. *Phys Rev Lett* 1997; **78:** 1396.

12 Weigend F and Ahlrichs R. Balanced basis sets of split valence, triple zeta valence and quadruple zeta valence quality for H to Rn: Design and assessment of accuracy. *Phys Chem Chem Phys* 2005; **7:** 3297-305.

13 Pollak P and Weigend F. Segmented contracted error-consistent basis sets of double- and triple-ζ valence quality for one- and two- component relativistic all-electron calculations. *J Chem Theory Comput* 2017; **13**: 3696-705.

14 Schleyer PvR, Maerker C and Dransfeld A *et al.* Nucleus-independent chemical shifts:  a simple and efficient aromaticity probe. *J Am Chem Soc* 1996; **118**: 6317-8.

15 Chen Z, Wannere CS and Corminboeuf C *et al.* Nucleus-Independent Chemical Shifts (NICS) as an aromaticity criterion. *Chem Rev* 2005; **105**: 3842-88.

16 Fallah-Bagher-Shaidaei H, Wannere CS and Corminboeuf C *et al.* Which NICS aromaticity index for planar π rings is best? *Org Lett* 2006; **8**: 863-6.
